# New 2-Methoxy Acetylenic Acids and Pyrazole Alkaloids from the Marine Sponge *Cinachyrella* sp.

**DOI:** 10.3390/md15110356

**Published:** 2017-11-11

**Authors:** Amin Mokhlesi, Rudolf Hartmann, Tibor Kurtán, Horst Weber, Wenhan Lin, Chaidir Chaidir, Werner E. G. Müller, Georgios Daletos, Peter Proksch

**Affiliations:** 1 Institute of Pharmaceutical Biology and Biotechnology, Heinrich Heine University, Universitätsstraße 1, 40225 Düsseldorf, Germany; amin.mokhlesi@uni-duesseldorf.de; 2 Department of Marine Biology, Faculty of Marine Sciences, Tarbiat Modares University, 46414-356 Noor, Iran; 3 Institute of Complex Systems: Strukturbiochemie, Forschungszentrum Jülich GmbH, ICS-6, 52425 Jülich, Germany; r.hartmann@fz-juelich.de; 4 Department of Organic Chemistry, University of Debrecen, 4002 Debrecen, Hungary; kurtan.tibor@science.unideb.hu; 5 Institute of Pharmaceutical and Medicinal Chemistry, Heinrich Heine University, Universitätsstraße 1, 40225 Düsseldorf, Germany; Horst.Weber@uni-duesseldorf.de; 6 State Key Laboratory of Natural and Biomimetic Drugs, Peking University, Health Science Center, 100191 Beijing, China; whlin@bjmu.edu.cn; 7 Center for Pharmaceutical and Medical Technology, Agency for the Assessment and Application Technology, 10340 Jakarta, Indonesia; chaidir@bppt.go.id; 8 Institute of Physiological Chemistry, University Medical Center of the Johannes Gutenberg University Mainz, 55128 Mainz, Germany; wmueller@uni-mainz.de

**Keywords:** natural products, marine sponge, *Cinachyrella* sp., 2-methoxy acetylenic acid, pyrazole alkaloid

## Abstract

Three new 2-methoxy acetylenic acids (**1**–**3**) and a known derivative (**4**), in addition to three new natural pyrazole alkaloids (**5**–**7**) were isolated from an Indonesian marine sponge of the genus *Cinachyrella*. Compounds **5** and **6** have previously been reported as synthetic compounds. The structures of the new compounds were established on the basis of one- and two-dimensional NMR spectroscopy as well as by mass spectrometric data. The absolute configuration of the new acetylenic acid derivatives (**1**–**3**) was established by ECD spectroscopy. All isolated compounds were evaluated for their cytotoxicity against L5178Y mouse lymphoma cells. Compounds **1**–**4** exhibited strong activity with an IC_50_ value of 0.3 µM. A plausible biosynthetic pathway for the pyrazole metabolites **5**–**7** is proposed.

## 1. Introduction

Marine sponges (phylum Porifera) are sessile filter feeder animals with the ability to produce bioactive secondary metabolites as an efficient defence mechanism against predators, fouling organisms or pathogenic microorganisms [1]. Several of these metabolites possess pronounced biological activities in disease relevant screens and have proven to be a valuable source of novel lead compounds in drug discovery. Prominent examples of drug leads based on marine natural products include halichondrin B (macrocyclic polyether; lead structure of anticancer drug Halaven^®^), dictyodendrins (pyrrolocarbazole derivatives; lead structures of a preclinical telomerase inhibitor), and sarcodictyin (diterpene; lead structure of a preclinical tubulin inhibitor) from the sponges *Halichondria okadai*, *Dictyodendrilla verongiformis*, and *Sarcodictyon roseum*, respectively [2].

Sponges of the genus *Cinachyrella* sp. are mostly known for the production of steroids [3] and fatty acid metabolites, such as the 2-methoxy acetylenic acid derivative cinachylenic acid A from *C. australiensis* [4], or long-chain isoprenoid fatty acids from *C. schulzei* [5,6]. In addition, sterols have been used in chemotaxonomic studies of *C. cavernosa* [7]. Notably, enigmazole A, an unprecedented phosphate-containing macrolide from the Papua New Guinea marine sponge *C. enigmatica*, has been reported to exhibit pronounced cytotoxicity against the NCI60 human tumour cell line and to interfere with c-Kit receptor signaling, a transmembrane glycoprotein with tyrosine kinase activity, which is involved in the growth and development of various cancers [8]. 

**Figure 1:**
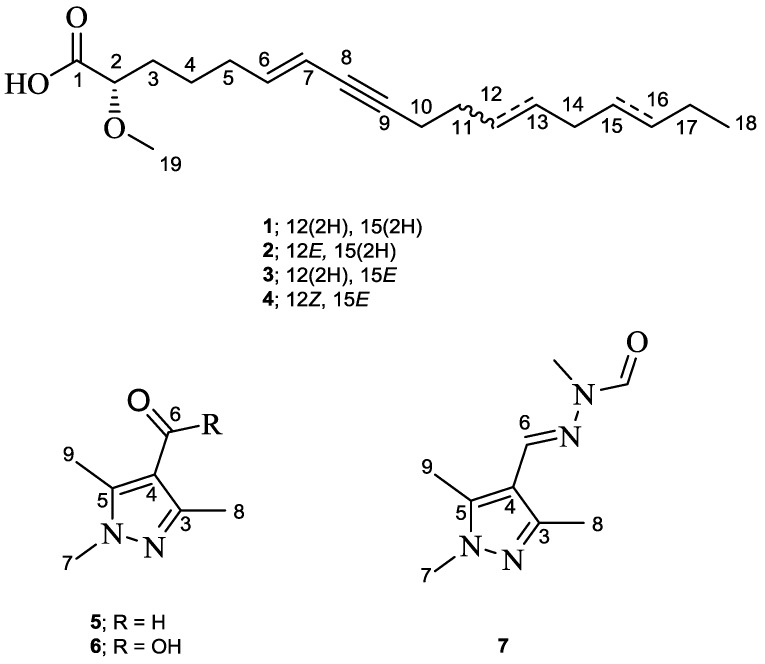
Structures of **1**–**7**.

As part of our ongoing search on bioactive secondary metabolites from marine sponges, we investigated a specimen of *Cinachyrella* sp. collected at Ambon Island in Indonesia. The MeOH extract exhibited significant activity against the murine lymphoma L5178Y cell line at a concentration of 10 μg/mL. Chromatographic separation of the extract afforded three new (**1**–**3**) acetylenic acid derivatives and one known (**4**) congener, in addition to three pyrazole alkaloids (**5**–**7**) (Figure 1). Herein, we report the structure elucidation and cytotoxic activity of the isolated compounds and provide a rationale for the biosynthesis of the pyrazole derivatives.

## 2. Results

### 2.1. Cinachylenic Acid B *(**1**)*

**Figure 2:**
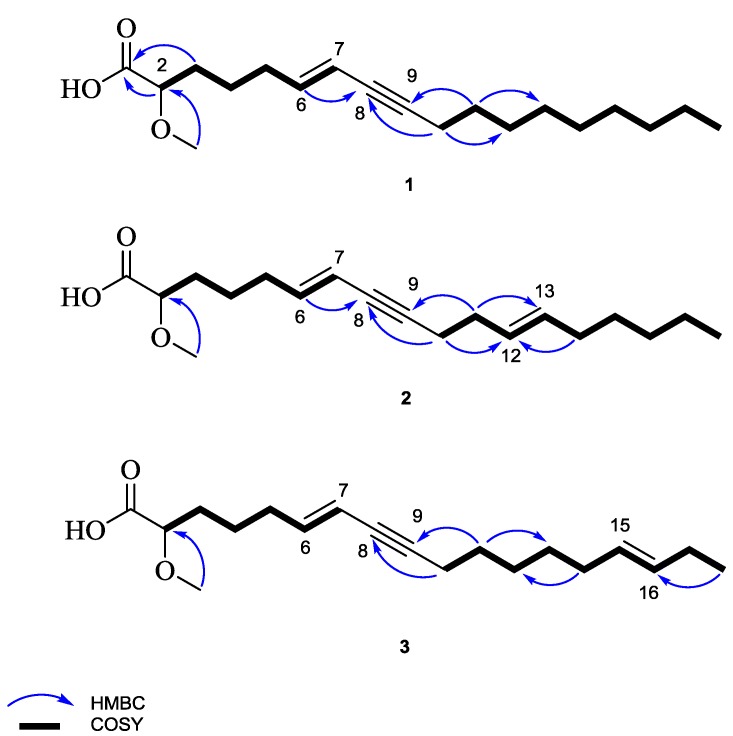
HMBC and COSY correlations of **1**–**3**.

Cinachylenic acid B (**1**) was obtained as yellow oil. The molecular formula was determined as C_19_H_32_O_3_ on the basis of the pseudomolecular ion peak at *m*/*z* 309.2420 [M + H]^+^ in the high-resolution electrospray ionization mass spectrometry (HRESIMS) spectrum indicating four degrees of unsaturation. In the ^1^H NMR spectrum, the fatty acid nature of **1** was apparent from the presence of a cluster of eleven methylene groups (δ_H_ 1.30–2.26) as well as a terminal methyl group at δ_H_ 0.90 (H_3_-18). Moreover, the ^1^H NMR spectrum revealed signals at δ_H_ 5.46 (H-7) and 5.95 (H-6) corresponding to an *E*-configurated disubstituted double bond on the basis of the large coupling constant (^3^*J* = 15.8 Hz), as well as a methoxy group and an oxygenated methine proton (H-2) based on their integration and low field resonances at δ_H_ 3.36 and 3.76, respectively (Table 1). The COSY spectrum of **1** showed two continuous spin systems corresponding to the fragments extending from H-2 to H-7 and from H_2_-10 to H_3_-18 (Figure 2). The HMBC spectrum confirmed the identified substructures and established their connection through key ^3^*J*-correlations from H-6 and H_2_-10 to C-8 (δ_c_ 79.9) and from H_2_-11 to C-9 (δ_c_ 89.2), denoting the presence of an alkyne group (C-8/9) in the structure of **1** (Figure 2). In addition, the correlation from H-2 and H_2_-3 to the ester carbonyl at δ_c_ 176.0 (C-1), suggested a terminal carboxylic acid group being located at C-2. Likewise, the remaining methoxy group (2-OCH_3_) was assigned at C-2, as confirmed by inspection of the respective HMBC correlation. Thus, the planar structure of **1** was established as shown in Figure 1. This assignment was further supported by EIMS, which showed fragmentation ions at *m*/*z* 178 and 263, presumably originating from cleavage at C-5/6 and C-1/2, respectively (Figure S1-5). Hence, compound **1** was identified as a new natural product and was named cinachylenic acid B.

### 2.2. Cinachylenic Acid C *(**2**)*

Compound **2** was isolated as yellow oil. The HRESIMS exhibited the pseudomolecular ion peak at *m*/*z* 307.2266 [M + H]^+^, consistent with the molecular formula C_19_H_30_O_3_, thus revealing five degrees of unsaturation. The ^1^H NMR spectrum of **2** was similar to that of **1**, apart from the replacement of the methylene groups H_2_-12 and H_2_-13 in **1** by two olefinic protons resonating at δ_H_ 5.45 and 5.48, respectively (Table 2). The aforementioned spectroscopic differences suggested that **2** features the same molecular framework as **1**, bearing an additional double bond (C-12/13), which is in agreement with the 2 amu molecular weight difference observed between both compounds. This assumption was supported by the HMBC correlations from H_2_-10 and H_2_-14 to C-12 (δ_c_ 129.8) and from H_2_-11 to C-13 (δ_c_ 132.3), as well as by the COSY cross-peaks between H_2_-11 (δ_H_ 2.16) and H-12, and between H_2_-14 (δ_H_ 2.00) and H-13 (Figure 2). In analogy to **1**, the structure of **2** was further corroborated by the EIMS fragment ion peaks at *m*/*z* 175 and 131, presumably originating from cleavage at C-5/6, as well as at *m*/*z* 209 and 97, from cleavage at C-11/12 (Figure S2-5). In addition, the geometry of the double bond C-12/13 was established as *E* based on the large coupling constant between H-12 and H-13 (^3^*J* = 15.3 Hz). Accordingly, compound **2** was identified as a new natural product and the name cinachylenic acid C is proposed.

### 2.3. Cinachylenic Acid D *(**3**)*

Compound **3** was isolated as yellow oil and shared the same molecular formula with **2** (C_19_H_30_O_3_), as indicated by the pseudomolecular ion peak at *m*/*z* 307.2264 [M + H]^+^ in the HRESIMS. In a similar manner to **2**, the ^1^H NMR data of **3** showed close similarity to those of **1** apart from the presence of an additional *E*-configured double-bond at C-15/16, as supported by the HMBC correlation from H_3_-18 to C-16 (δ_C_ 132.9) and by the large coupling constant between H-15 and H-16 (^3^*J* = 15.3 Hz) (Table 3). Similarly, the structure of **3** was further corroborated by the EIMS fragment ion peaks at *m*/*z* 149, 175 and 278, presumably originating from cleavage at C-7/8, C-5/6 and C-16/17, respectively (Figure S3-5). Thus, **3** was characterized as the double-bond positional isomer of **2** and was named cinachylenic acid D.

### 2.4. Cinachylenic Acid A *(**4**)*

Compound **4** was identified as 2-methoxy-trien-8-yne-octadecanoic acid, originally reported from the marine sponge *C. australiensis*, based on its NMR and HRESIMS data and by comparison with the literature [4]. The trivial name cinachylenic acid A is proposed.

### 2.5. Absolute Configuration of Cinachylenic Acids A–D *(**1**–**4**)*

The experimental ECD spectra of **1**–**3** were rather weak, suggesting that these compounds were obtained as enantiomeric mixtures. Nevertheless, their ECD spectra showed a positive Cotton effect at around 210 nm (Figures S1-6, S2-6, and S3-6), which could be attributed to the low enantiomeric excess of the (*S*) enantiomer, as previously established for 2-methoxy acetylenes and fatty acids [9]. It should be noted that the ECD spectrum of **4** was not obtained due to its rapid decomposition during storage. However, the stereocenter C-2 in **4** was assumed to be (*S*) based on its specific rotation value, which has a negative sign, similarly to those of **1**–**3**, as well as its close biogenetic relationship with the latter metabolites.

### 2.6. Cinachyrazole A *(**5**)*

**Figure 3:**
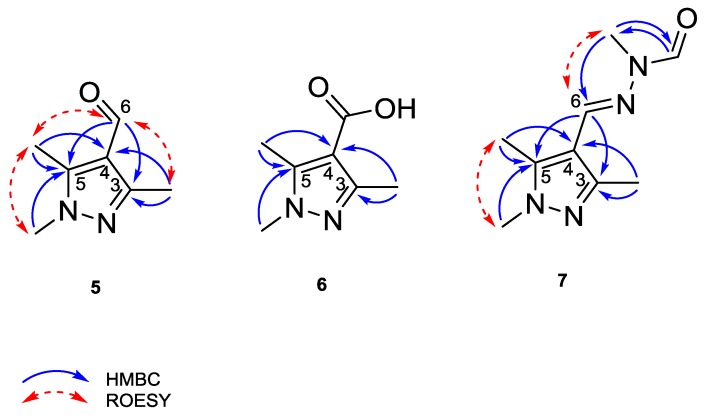
HMBC and ROESY correlations of **5**–**7**.

Compound **5** was isolated as a white, amorphous solid. Its molecular formula was established as C_7_H_10_N_2_O based on the pseudomolecular ion peak observed at *m*/*z* 139.0865 [M + H]^+^ in the HRESIMS spectrum, corresponding to four degrees of unsaturation. Inspection of the ^1^H NMR spectrum indicated the presence of three methyl groups at δ_H_ 3.74 (H_3_-7), 2.38 (H_3_-8), and 2.51 (H_3_-9), in addition to a singlet proton at δ_H_ 9.85 (H-6) characteristic of an aldehyde group. Analysis of the HMBC spectrum confirmed the corresponding carbon signals and revealed in addition three sp^2^ quaternary carbon atoms at δ_C_ 151.7 (C-3), 119.0 (C-4), and 147.1 (C-5) (Table 4). HMBC correlations from H_3_-8 to C-3 and C-4, from H_3_-9 to C-5 and C-4, and from H_3_-7 to C-5 were indicative of a 1,3,5-trimethylpyrazole ring. The remaining aldehyde group was attached to C-4 based on the observed HMBC correlations from H-6 to C-5 and C-3 (Figure 3). This structural assignment was further supported by the ROESY cross-peaks between H_3_-7 and H_3_-9 as well as between H-6 and both H_3_-8 and H_3_-9. Hence, compound **5** was identified as a new natural product and was named cinachyrazole A.

### 2.7. Cinachyrazole B *(**6**)*

Compound **6** was isolated as a white, amorphous solid. Its molecular formula was determined as C_7_H_10_N_2_O_2_, in accordance with the pseudomolecular ion peak observed at *m*/*z* 155.0814 [M + H]^+^ in the HRESIMS spectrum. The ^1^H NMR data of **6** were almost superimposable to those of **5**, except for the absence of the aldehyde group signal at δ_H_ 9.85 (H-6 in **5**). This spectral difference suggested that **6** features the same pyrazole core structure as **5**, apart from the replacement of the aldehyde group by a carboxylic acid group, which is in accordance with the 16 amu increase in the molecular weight compared to **5** (Table 4). This assumption was further corroborated by analysis of the HMBC spectrum of **6** (Figure 3). Accordingly, the structure of **6** was assigned and the compound was named cinachyrazole B.

### 2.8. Cinachyrazole C *(**7**)*

Compound **7** was isolated as a white, amorphous solid. The HRESIMS of **7** exhibited a pseudomolecular ion peak at *m*/*z* 195.1241 [M + H]^+^, consistent with the molecular formula C_9_H_14_N_4_O. The ^1^H and ^13^C NMR data of **7** denoted the same pyrazole basic unit as in **5** and **6** (Table 4). The remaining resonances included an olefinic signal at δ_H_ 7.95 (CH-6, δ_c_ 138.0), an *N*-methyl group at δ_H_ 3.29 (*N*′-CH_3_, δ_c_ 26.4), and an aldehyde group at δ_H_ 8.69 (*N*′-CHO, δ_c_ 166.1). These signals were indicative of the presence of an *N’*-formyl-*N’*-methyl-hydrazone moiety at C-4, as supported by the HMBC correlations from H-6 to C-5 and C-3 and from H_3_-*N*′-CH_3_ to *N*′-CHO. In addition, the *E* geometry of the double bond (C-6/N) was established based on the observed ROESY cross-peak between H-6 and H_3_-N′-CH_3_ (Figure 3). For compound **7**, the name cinachyrazole C is suggested.

### 2.9. Proposed Biosynthetic Pathway for ***5**–**7***

**Figure 4:**
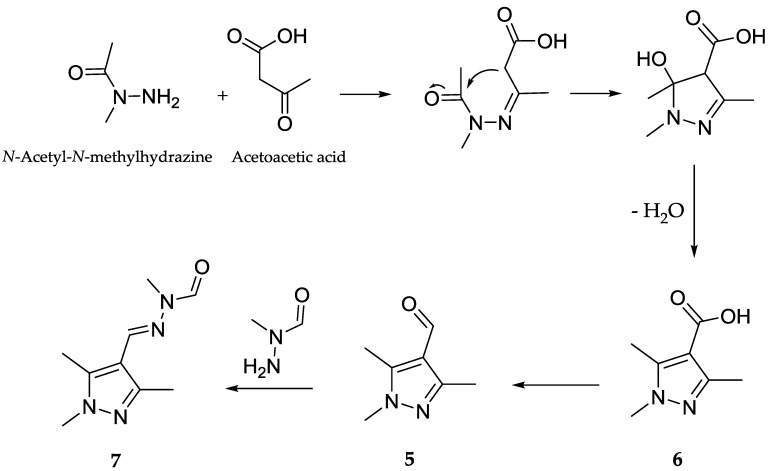
Proposed biosynthesis of **5**–**7**.

A plausible biosynthetic pathway for the isolated pyrazole derivatives **5**–**7** is proposed, which includes the linkage of an acetoacetic acid and an *N*-acetyl-*N*-methylhydrazine unit via a Schiff base formation followed by cyclization and dehydration reactions to form **6**. Subsequent reduction of the latter followed by a Schiff base formation with an *N*-formyl-*N*-methylhydrazine unit, a natural product originally isolated from the ascomycete fungus *Gyromitra esculenta* [10], would finally result in the formation of **5** and **7**, respectively (Figure 4).

### 2.10. Bioactivity

All isolated compounds (**1**–**7**) were evaluated for their effects on the growth of the L5178Y mouse lymphoma cell line employing the MTT assay. Interestingly, the acetylenic acid derivatives **1**–**4** exhibited pronounced cytotoxicity with an IC_50_ value of 0.3 µM compared to the positive control kahalalide F (IC_50_ = 4.3 µM). The pyrazole derivatives (**5**–**7**) showed no cytotoxicity in the respective assay (IC_50_ > 10 µM).

## 3. Discussion

Compounds **1**–**4** are acetylenic acid derivatives possessing a methoxy group at C-2 as well as an acetylene group at C-8/9; however, they differ in the number and/or position of double bonds at their aliphatic chain (Figure 1). Other examples of 2-methoxy acetylenic acids from marine sponges include corticatic acids A–E, isolated from sponge *Petrosia corticata* with antifungal activity [11,12], taurospongin A from the sponge *Hippospongia* sp. with inhibitory activity against DNA polymerase *β* and HIV reverse transcriptase [13], as well as halicynones A and B from the sponge *Haliclona* sp. with antitumor activity against the human colon tumor cell line (HCT-116) [14]. Compounds **5**–**7** possess an unusual 1,3,5-trimethylpyrazole core structure. It should be noted that **5** and **6** have been previously described as synthetic compounds and this is the first report of their isolation as natural products (Tables S5-8 and S6-6) [15,16]. Interestingly, compound **7** bears an additional hydrazone unit, which is rarely reported from marine invertebrates [11]. To the best of our knowledge, in addition to **7**, the only examples so far of sponge-derived compounds bearing a hydrazone unit include cinachyramine, likewise from a marine sponge of the genus *Cinachyrella* [12], as well as psammaplin G, a potent inhibitor of DNA methyltransferase, from the sponge *Pseudoceratina purpurea* [14].

## 4. Materials and Methods

### 4.1. General Procedures

1D and 2D NMR spectra were recorded on an AVANCE III HD 600 NMR spectrometer (Bruker, Karlsruhe, Germany) and the chemical shifts were referenced to the solvent residual peaks. HRESIMS and EIMS were measured on a UHR-QTOF maxi 4G (Bruker Daltonics, Bremen, Germany) or on a Thermo Finnigan TCQ 7000 mass spectrometer (Thermo Fisher Scientific GmbH, Bremen, Germany), respectively. UV data were recorded on a Perkin-Elmer Lambda 25 UV/vis spectrometer (Perkin-Elmer, Waltham, MA, USA). ECD spectra were recorded with a J-810 spectropolarimeter (JASCO International Co. Ltd, Tokyo, Japan). The HPLC analysis was carried out using a Dionex UltiMate-3400SD equipped with a LPG-3400SD pump and a DAD 300RS photodiode array detector (Dionex Softron GmbH, Munich, Germany). The analytical column was filled with Eurosphere-10 C18 (125 × 4 mm, L × i.d.) (Knauer, Berlin, Germany), and the gradient consisted of 0 min (10% MeOH); 5 min (10% MeOH); 35 min (100% MeOH); 45 min (100% MeOH), with MeOH, 0.1% formic acid in H_2_O solvent system. UV detection during HPLC was set at 235, 254, 280, and 340 nm. A Merck Hitachi HPLC System (UV detector L-7400; pump L-7100; Eurosphere-100 C18, 300 mm × 8 mm, Knauer, Berlin, Germany), was used for semipreparative HPLC with a gradient system of MeOH/H_2_O as mobile phase. Column chromatography was performed using Sephadex LH-20 (GE Healthcare Europe GmbH, Freiburg, Germany) and silica gel 60 M (0.04–0.063 mm) (Macherey-Nagel, Düren, Germany) as stationary phases. Thin-layer chromatography (TLC) was run using pre-coated silica gel 60 F254 plates (Merck KGaA, Darmstadt, Germany) followed by UV detection at 254 nm or after spraying the plates with anisaldehyde reagent. Optical rotations were measured using a Jasco P-2000 polarimeter (JASCO International Co., Ltd., Tokyo, Japan).

### 4.2. Sponge Material 

The sponge specimen was collected by scuba at Ambon, Indonesia and was identified as *Cinachyrella* sp. by Dr. Nicole de Voogd (Naturalis Biodiversity Center, Leiden, The Netherlands). A voucher specimen was deposited at the Naturalis Biodiversity Center under the reference number RMNH POR. 10903. The sponge specimen was preserved in a mixture of EtOH and H_2_O (70:30) and stored in a freezer (−20 °C) prior to extraction.

### 4.3. Extraction and Isolation 

The sponge (wet weight 400 g) was cut into small pieces. The material was exhaustively extracted with methanol (3 × 1 L) at room temperature and concentrated under vacuum to yield 1.4 g dry crude extract. The obtained extract was submitted to liquid-liquid partitioning to afford *n*-hexane, ethyl acetate and *n*-butanol fractions. The resulting fractions were further purified by column chromatography on Sephadex LH-20 (MeOH as mobile phase) and/or by vacuum liquid chromatography (VLC) using *n*-hexane/ethyl acetate and DCM/MeOH step gradient elution, followed by semipreparative HPLC, in a gradient system of H_2_O/MeOH, to yield **1** (1 mg), **2** (1.3 mg), **3** (0.8 mg), and **4** (1 mg) from *n*-hexan, as well as **5** (2.5 mg), **6** (2.0 mg), and **7** (1.0 mg) from ethyl acetate fraction.

Cinachylenic acid B (**1**): Yellow oil; [α${]}_{D}^{20}$ = −11 (*c* 0.1, MeOH); UV (λ_max_, MeOH) (log ε) 229 (4.1); HRESIMS *m*/*z* 309.2420 [M + H]^+^ (calcd. for C_19_H_33_O_3_, 309.2424); EIMS *m*/*z* (relative intensity %) 263 (32), 178 (26); ^1^H and ^13^C NMR spectroscopic data, see Table 1.

Cinachylenic acid C (**2**): Yellow oil; [α${]}_{D}^{20}$ = −30 (*c* 0.1, MeOH); UV (λ_max_, MeOH) (log ε) 228 (4.0); HRESIMS *m*/*z* 307.2266 [M + H]^+^ (calcd. for C_19_H_31_O_3_, 307.2268); EIMS *m*/*z* (relative intensity %) 209 (4), 175 (100), 131 (70), 97 (28); ^1^H and ^13^C NMR spectroscopic data, see Table 2.

Cinachylenic acid D (**3**): Yellow oil; [α${]}_{D}^{20}$ = −20 (*c* 0.1, MeOH); UV (λ_max_, MeOH) (log ε) 228 (3.9); HRESIMS *m*/*z* 307.2264 [M + H]^+^ (calcd. for C_19_H_31_O_3_, 307.2268); EIMS *m*/*z* (relative intensity %) 278 (15), 175 (12), 149 (78), 131 (17); ^1^H and ^13^C NMR spectroscopic data, see Table 3.

Cinachyrazole A (**5**): White, amorphous solid; UV (λ_max_, MeOH) (log ε) 201 (3.3), 256 (3.8); HRESIMS *m*/*z* 139.0865 [M + H]^+^ (calcd. for C_7_H_11_N_2_O, 139.0866); ^1^H and ^13^C NMR spectroscopic data, see Table 4.

Cinachyrazole B (**6**): White, amorphous solid; UV (λ_max_, MeOH) (log ε) 229 (3.5); HRESIMS *m*/*z* 155.0814 [M + H]^+^ (calcd. for C_7_H_11_N_2_O_2_ 155.0815); ^1^H and ^13^C NMR spectroscopic data, see Table 4.

Cinachyrazole C (**7**): White, amorphous solid; UV (λ_max_, MeOH) (log ε) 266 (3.8), 288 (4.1); HRESIMS *m*/*z* 195.1241 [M + H]^+^ (calcd. for C_9_H_15_N_4_O, 195.1240); ^1^H and ^13^C NMR spectroscopic data, see Table 4.

## 5. Conclusions

In summary, a chemical investigation of the marine sponge *Cinachyrella* sp. collected in Indonesia afforded three new acetylenic acid derivatives (**1**–**3**) and one known congener (**4**), in addition to three new pyrazole alkaloids (**5**–**7**). The unusual pyrazole ring of the latter is postulated to be derived via a Schiff base formation between an acetoacetic acid and an *N*-acetyl-*N*-methylhydrazine unit. Notably, the acetylenic acid derivatives **1**–**4** showed strong inhibitory effect on the growth of L5178Y mouse lymphoma cell line with an IC_50_ value of 0.3 µM. In this context, our report highlights the metabolic potential of marine sponges of the genus *Cinachyrella* as a rich source of novel bioactive secondary metabolites.

## Figures and Tables

**Table 1:** ^1^H (600 MHz), ^13^C (150 MHz), COSY, and HMBC data of cinachylenic acid B (**1**) in MeOH-*d*_4_, δ in ppm.

Position	^13^C, Type ^1^	^1^H, m (*J* in Hz)	COSY	HMBC
1	176.0, C	- ^3^	- ^3^	- ^3^
2	80.9, CH	3.76, dd (7.6, 4.6)	3-H	C-1/3/4/19
3	32.8, CH_2_	1.74, m; 1.68, m	2/4-H	C-1/2/4/5
4	25.3, CH_2_	1.50 ^2^	3/5-H	C-2/5/6
5	33.1, CH_2_	2.10, br qt (7.2, 1.9)	4/6-H	C-3/4/6/7
6	142.7, CH	5.95, dt (15.8, 7.1)	5/7-H	C-5/8
7	111.4, CH	5.46, dt (15.8, 1.9)	6-H	- ^3^
8	79.9, C	- ^3^	- ^3^	- ^3^
9	89.2, C	- ^3^	- ^3^	- ^3^
10	19.6, CH_2_	2.26, br td (7.0, 2.1)	11-H	C-7/8/9/11/12
11	29.5, CH_2_	1.49 ^2^; 1.40, m	10/12-H	C-9/10/13
12	29.5, CH_2_	1.30 ^2^	11/13-H	- ^3^
13	30.4, CH_2_	1.30 ^2^	12/14-H	- ^3^
14	30.4, CH_2_	1.30 ^2^	13/15-H	- ^3^
15	30.4, CH_2_	1.30 ^2^	14/16-H	- ^3^
16	32.9, CH_2_	1.30 ^2^	15/17-H	- ^3^
17	23.5, CH_2_	1.33 ^2^	16/18-H	C-16/18
18	14.0, CH_3_	0.90, t (7.0)	17-H	C-16/17
19	57.9, CH_3_	3.36, s	- ^3^	C-2

^1^ Data extracted from HMBC spectra, ^2^ Overlapped signals, ^3^ None.

**Table 2:** ^1^H (600 MHz), ^13^C (150 MHz), COSY, and HMBC data of cinachylenic acid C (**2**) in MeOH-*d*_4_, δ in ppm.

Position	^13^C, Type ^1^	^1^H, m (*J* in Hz)	COSY	HMBC
1	– ^2^	- ^3^	- ^3^	- ^3^
2	80.9, CH	3.76, dd (7.6, 4.6)	3-H	C-4/5/19
3	32.8, CH_2_	1.74, m; 1.68, m	2/4-H	- ^3^
4	25.3, CH_2_	1.50, m	3/5-H	C-2/5/6
5	33.1, CH_2_	2.10, br qt (7.3, 2.0)	4/6-H	C-3/4/6/7
6	142.8, CH	5.95, dt (15.7, 7.3)	5/7-H	C-8
7	111.4, CH	5.46, d (15.7)	6-H	- ^3^
8	80.0, C	- ^3^	- ^3^	- ^3^
9	88.6, C	- ^3^	- ^3^	- ^3^
10	20.6, CH_2_	2.28, td (7.1, 2.1)	11-H	C-8/9/11/12
11	32.7, CH_2_	2.16, br q (6.5)	10/12-H	C-9/12/13
12	129.8, CH	5.45 dt (15.3, 6.5)	11/13-H	- ^3^
13	132.3, CH	5.48 dt (15.3, 6.0)	12/14-H	- ^3^
14	32.5, CH_2_	2.00, br q (6.0)	13/15-H	C-12/13/16
15	30.4, CH_2_	1.36, m	14/16-H	- ^3^
16	31.9, CH_2_	1.30, m	15/17-H	C-15
17	23.3, CH_2_	1.33, m	16/18-H	C-16
18	14.0, CH_3_	0.90, t (6.9)	17-H	C-16/17
19	58.0, CH_3_	3.36, s	- ^3^	C-2

^1^ Data extracted from HMBC spectra, ^2^ Not observed, ^3^ None.

**Table 3:** ^1^H (600 MHz), ^13^C (150 MHz), COSY, and HMBC data of cinachylenic acid D (**3**) in MeOH-*d*_4_, δ in ppm.

Position	^13^C, Type ^1^	^1^H, m (*J* in Hz)	COSY	HMBC
1	– ^2^	- ^4^	- ^4^	- ^4^
2	81.2, CH	3.74, dd (8.1, 4.7)	3-H	
3	32.6, CH_2_	1.74, m; 1.67, m	2/4-H	C-2/4/5
4	25.4, CH_2_	1.50 ^3^	3/5-H	C-2/5/6
5	33.2, CH_2_	2.10, br qt (7.2, 1.9)	4/6-H	C-3/4/6/7
6	142.8, CH	5.95, dt (15.9, 7.1)	5/7-H	C-5/8
7	111.4, CH	5.46, d (15.9)	6-H	- ^4^
8	79.8, C	- ^4^	- ^4^	- ^4^
9	88.9, C	- ^4^	- ^4^	- ^4^
10	19.5, CH_2_	2.25, td (7.0, 2.2)	11-H	C-8/9/11/12
11	29.8, CH_2_	1.49 ^3^	10/12-H	C-9/10
12	29.1, CH_2_	1.30, m	11/13-H	- ^4^
13	29.9, CH_2_	1.38, m	12/14-H	C-14
14	30.3, CH_2_	1.99 ^3^	13/15-H	C-12/13
15	– ^2^	5.43 ^3^	14/16-H	- ^4^
16	132.9, CH	5.39, dt (15.3, 6.3)	15/17-H	- ^4^
17	26.3, CH_2_	1.99 ^3^	16/18-H	C-18
18	14.2, CH_3_	0.96, t (7.5)	17-H	C-16/17
19	58.0, CH_3_	3.36, s	- ^4^	C-2

^1^ Data extracted from HMBC spectra, ^2^ Not observed, ^3^ Overlapped signals, ^4^ None.

**Table 4:** ^1^H NMR (600 MHz) and ^13^C NMR (150 MHz) data of **5**, **6**, and **7** in MeOH-*d*_4_, δ in ppm.

Position	5		6		7	
	^1^H, m	^13^C, Type ^1^	^1^H, m	^13^C, Type ^1^	^1^H, m	^13^C, Type ^1^
3	- ^3^	151.7, C	- ^3^	151.2, C	- ^3^	147.9, C
4	- ^3^	119.0, C	- ^3^	110.5, C	- ^3^	113.1, C
5	- ^3^	147.1, C	- ^3^	145.7, C	- ^3^	141.4, C
6	9.85, s	186.8, CH	- ^3^	– ^2^	7.95, s	138.0, CH
7	3.74, s	35.7, CH_3_	3.72, s	35.8, CH_3_	3.73, s	35.5, CH_3_
8	2.38, s	12.4, CH_3_	2.35, s	– ^2^	2.35, s	13.0, CH_3_
9	2.51, s	9.7, CH_3_	2.50, s	11.5, CH_3_	2.45, s	9.9, CH_3_
*N*′-CHO	- ^3^	- ^3^	- ^3^	- ^3^	8.69, s	166.1, CH
*N*′-CH_3_	- ^3^	- ^3^	- ^3^	- ^3^	3.29, s	26.4, CH_3_

^1^ Data extracted from HMBC spectra, ^2^ Not observed, ^3^ None.

## Supplementary Materials

The following are available online at http://www.mdpi.com/1660-3397/15/11/356/s1, Supplementary Materials including HPLC, UV, HRESIMS, EIMS, 1D and 2D NMR spectra of **1**–**7**.
