# Supplementary material for: New 2-Methoxy Acetylenic Acids and Pyrazole Alkaloids from the Marine Sponge Cinachyrella sp
Source: Mar Drugs. 2017 Nov 11;15(11):356. doi: 10.3390/md15110356 (PMC5706045; doi:10.3390/md15110356)
Supplement: Supplementary file 1 [file marinedrugs-15-00356-s001.pdf]

Supplementary material

New 2-Methoxy Acetylenic Acids and Pyrazole Alkaloids from the Marine Sponge *Cinachyrella* sp.

Amin Mokhlesi [1,2], Rudolf Hartmann [3], Tibor Kurtán [4], Horst Weber [5], Wenhan Lin [6], Chaidir Chaidir [7], Werner E. G. Müller [8], Georgios Daletos [1,*], and Peter Proksch [1,*]

[1] Institute of Pharmaceutical Biology and Biotechnology, Heinrich Heine University, Universitätsstraße 1, 40225 Düsseldorf, Germany; amin.mokhlesi@uni-duesseldorf.de

[2] Department of Marine Biology, Faculty of Marine Sciences, Tarbiat Modares University, 46414-356 Noor, Iran

[3] Institute of Complex Systems: Strukturbiochemie, Forschungszentrum Jülich GmbH, ICS-6, 52425 Jülich, Germany; r.hartmann@fz-juelich.de

[4] Department of Organic Chemistry, University of Debrecen, 4002 Debrecen, Hungary; kurtan.tibor@science.unideb.hu

[5] Institute of Pharmaceutical and Medicinal Chemistry, Heinrich Heine University, Universitätsstraße 1, 40225 Düsseldorf, Germany; Horst.Weber@uni-duesseldorf.de

[6] State Key Laboratory of Natural and Biomimetic Drugs, Peking University, Health Science Center, 100191 Beijing, China; whlin@bjmu.edu.cn

[7] Center for Pharmaceutical and Medical Technology, Agency for the Assessment and Application Technology, 10340 Jakarta, Indonesia; chaidir@bppt.go.id

[8] Institute of Physiological Chemistry, University Medical Center of the Johannes Gutenberg University Mainz, 55128 Mainz, Germany; wmueller@uni-mainz.de

* Correspondence: georgios.daletos@uni-duesseldorf.de (G.D.); proksch@uni-duesseldorf.de (P.P.); Tel.: +49-211-81-14173 (G.D.); +49-211-81-14163 (P.P.)

Table of Contents

S1-1. ^{1}H NMR (MeOH-d_4, 600 MHz) spectrum of 1 5

S1-2. COSY NMR (MeOH-d_4, 600 MHz) spectrum of 1 6

S1-3. HMBC NMR (MeOH-d_4, 600 MHz) spectrum of 1 7

S1-4. HRESIMS spectrum of 1 8

S1-5. EIMS spectrum of 1 9

S1-6. CD spectrum of 1 10

S1-7. HPLC chromatogram of 1 11

S1-8. UV spectrum of 1 11

S2. Cinachylenic acid C (2) 12

S2-1. ^{1}H NMR (MeOH-d_4, 600 MHz) spectrum of 2 12

S2-2. COSY NMR (MeOH-d_4, 600 MHz) spectrum of 2 13

S2-3. HMBC NMR (MeOH-d_4, 600 MHz) spectrum of 2 14

S2-4. HRESIMS spectrum of 2 15

S2-5. EIMS spectrum of 2 16

S2-6. CD spectrum of 2 17

S2-7. HPLC chromatogram of 2 18

S2-8. UV spectrum of 2 18

S3. Cinachylenic acid D (3) 19

S3-1. ^{1}H NMR (MeOH-d_4, 600 MHz) spectrum of 3 19

S3-2. COSY NMR (MeOH-d_4, 600 MHz) spectrum of 3 20

S3-3. HMBC NMR (MeOH-d_4, 600 MHz) spectrum of 3 21

S3-4. HRESIMS spectrum of 3 22

S3-5. EIMS spectrum of 3 23

S3-6. CD spectrum of 3 24

S3-7. HPLC chromatogram of 3 25

S3-8. UV spectrum of 3 25

S4. Cinachylenic acid A (4) 26
S4-1. ^{1}H NMR (MeOH-d_4, 600 MHz) spectrum of 4 26
S4-2. HRESIMS spectrum of 4 27
S4-3. HPLC chromatogram of 4 28
S4-4. UV spectrum of 4 28
S5. Cinachyrazole A (5) 29
S5-1. ^{1}H NMR (MeOH-d_4, 600 MHz) spectrum of 5 29
S5-2. HMBC NMR (MeOH-d_4, 600 MHz) spectrum of 5 30
S5-3. ROESY NMR (MeOH-d_4, 600 MHz) spectrum of 5 31
S5-4. HRESIMS spectrum of 5 32
S5-5. HPLC chromatogram of 5 33
S5-6. UV spectrum of 5 33
S5-7. Comparison of UV data of 5 with those reported in the literature 33
S5-8. Comparison of ^{1}H NMR (600 MHz) data of 5 with those reported in the literature 34
S6. Cinachyrazole B (6) 35
S6-1. ^{1}H NMR (MeOH-d_4, 600 MHz) spectrum of 6 35
S6-2. HMBC NMR (MeOH-d_4, 600 MHz) spectrum of 6 36
S6-3. HRESIMS spectrum of 6 37
S6-4. HPLC chromatogram of 6 38
S6-5. UV spectrum of 6 38
S6-6. Comparison of the ^{13}C NMR (150 MHz) data of 6 with those reported in the literature 39
S7. Cinachyrazole C (7) 40
S7-1. ^{1}H NMR (MeOH-d_4, 600 MHz) spectrum of 7 40
S7-2. COSY NMR (MeOH-d_4, 600 MHz) spectrum of 7 41
S7-3. HMBC NMR (MeOH-d_4, 600 MHz) spectrum of 7 42
S7-4. ROESY NMR (MeOH-d_4, 600 MHz) spectrum of 7 43
S7-5. HRESIMS spectrum of 7 44

S7-6. HPLC chromatogram of 7 45

S7-7. UV spectrum of 7 45

S1. Cinachylenic acid B (**1**)

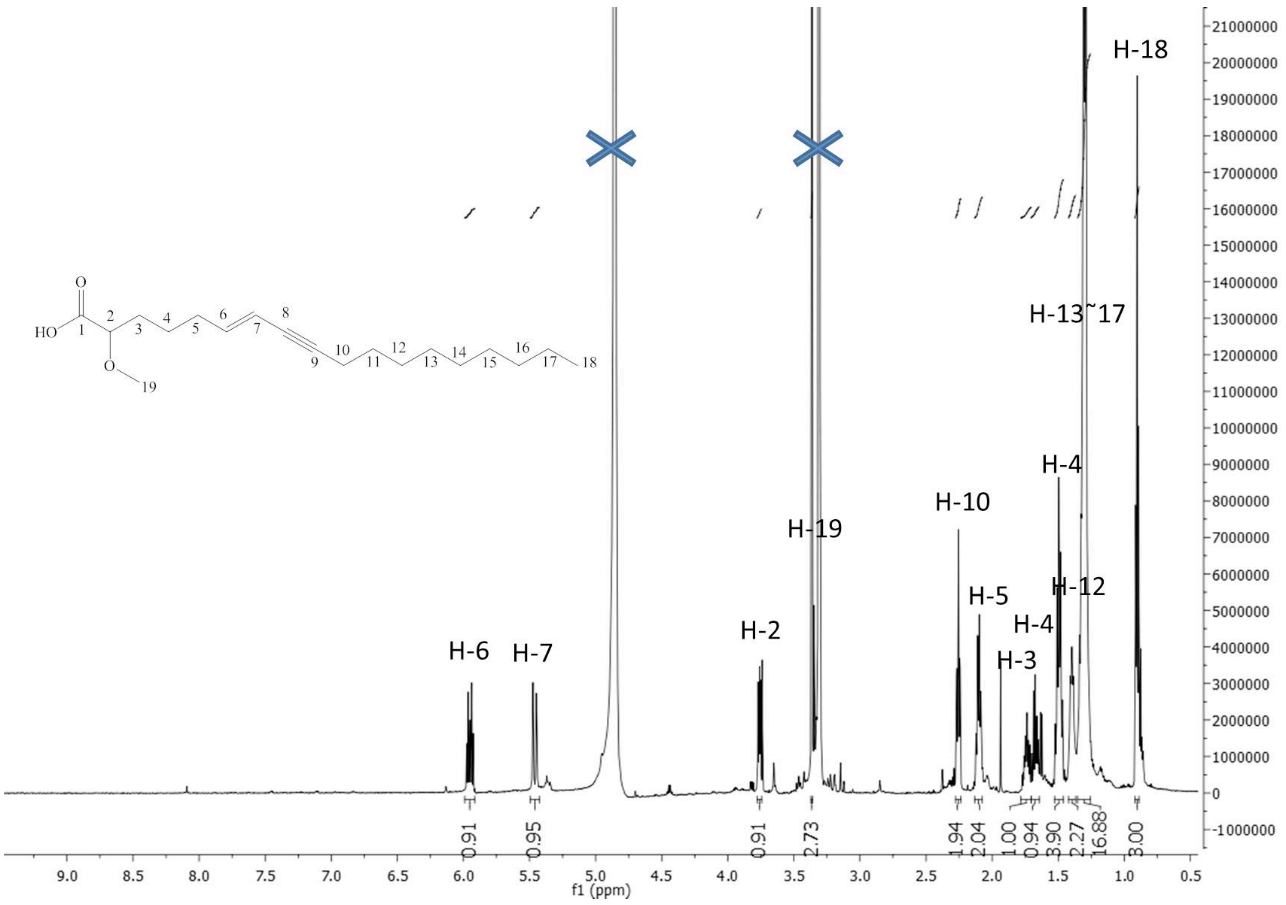

S1-1. ^{1}H NMR (MeOH-*d*$_4$, 600 MHz) spectrum of **1**

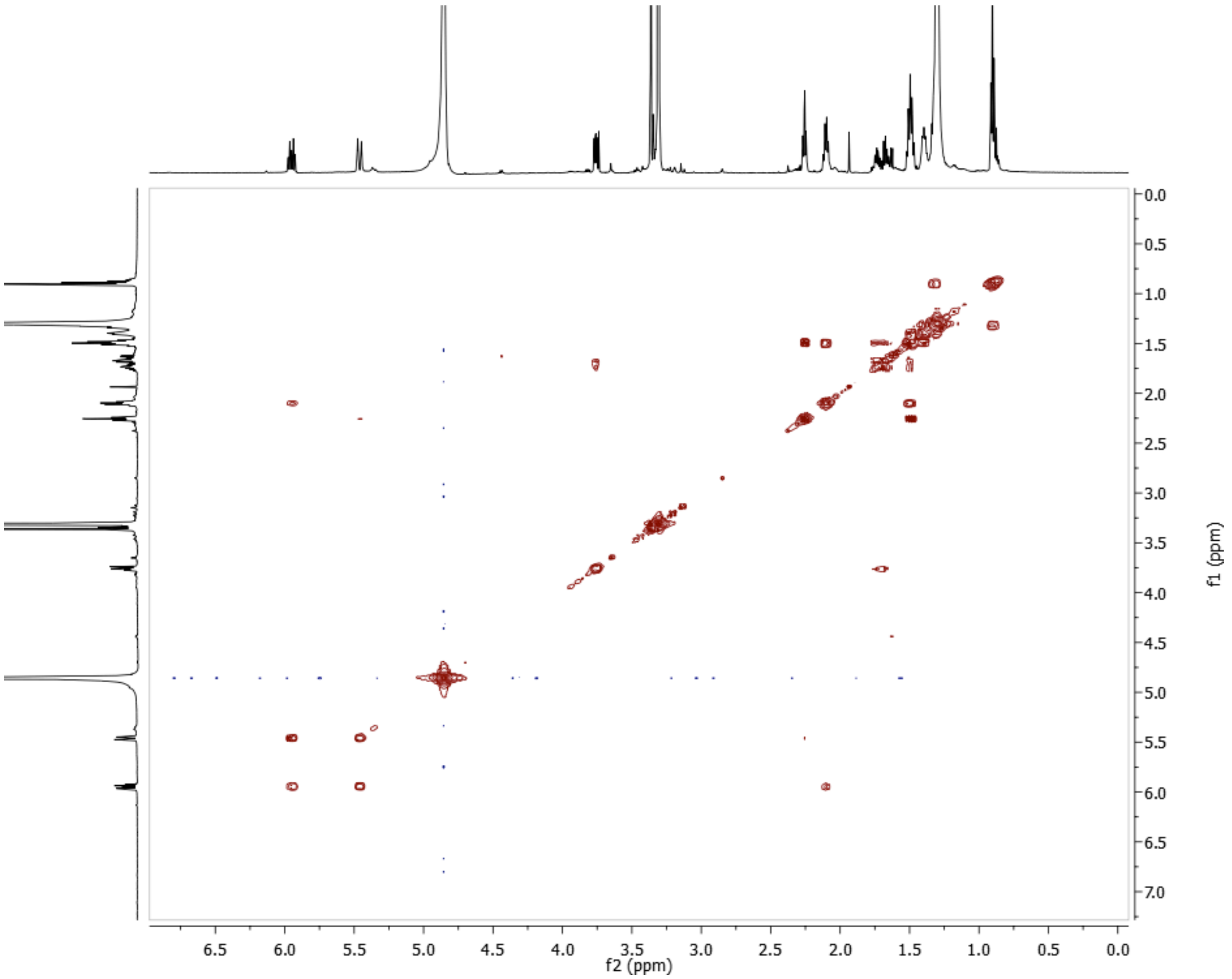

S1-2. COSY NMR (MeOH-*d*$_4$, 600 MHz) spectrum of **1**

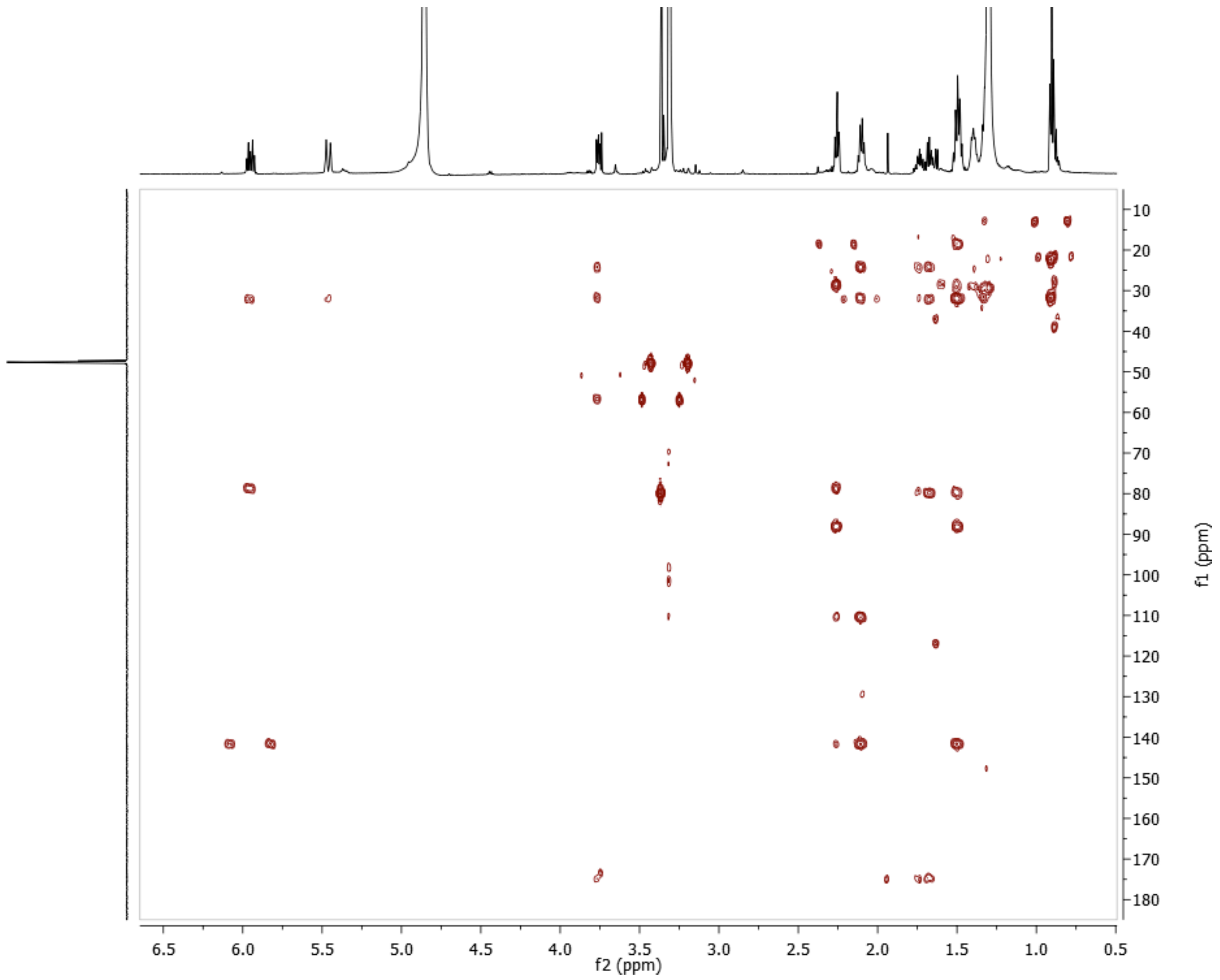

S1-3. HMBC NMR (MeOH-d_4, 600 MHz) spectrum of **1**

Mass Spectrum SmartFormula Report

Analysis Info

Analysis Name	D:\Data\spektren 2016\Proksch16HR000179.d	Acquisition Date	7/7/2016 2:42:36 PM
Method	tune_low_new.m	Operator	Peter Tommes
Sample Name	Amin Mokhlesi C2 E3-2-4 (CH3OH)	Instrument	maXis 288882.20213
Comment			

Acquisition Parameter

Source Type	ESI	Ion Polarity	Positive	Set Nebulizer	0.3 Bar
Focus	Not active	Set Capillary	4000 V	Set Dry Heater	180 °C
Scan Begin	50 m/z	Set End Plate Offset	-500 V	Set Dry Gas	4.0 l/min
Scan End	1500 m/z	Set Collision Cell RF	600.0 Vpp	Set Divert Valve	Source

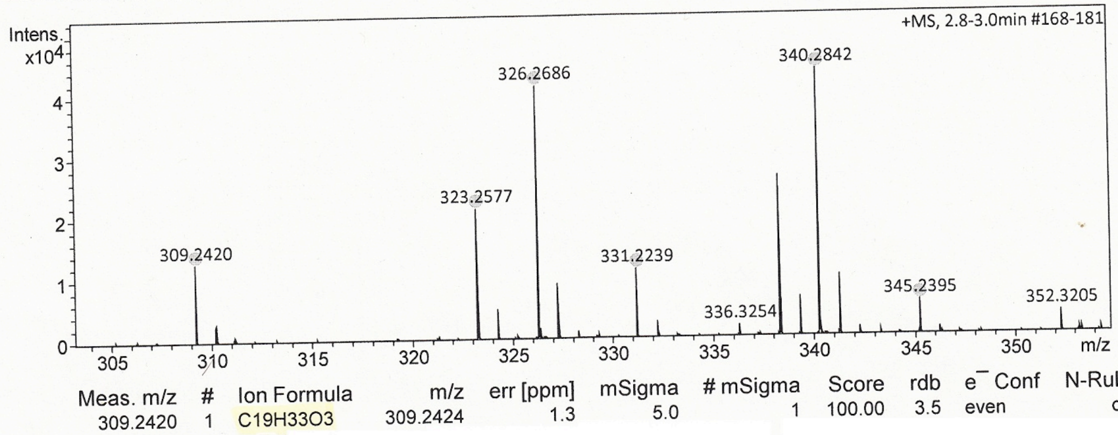

Meas. m/z	#	Ion Formula	m/z	err [ppm]	mSigma	# mSigma	Score	rdb	e⁻ Conf	N-Rule
309.2420	1	C19H33O3	309.2424	1.3	5.0	1	100.00	3.5	even	ok

S1-4. HRESIMS spectrum of **1**

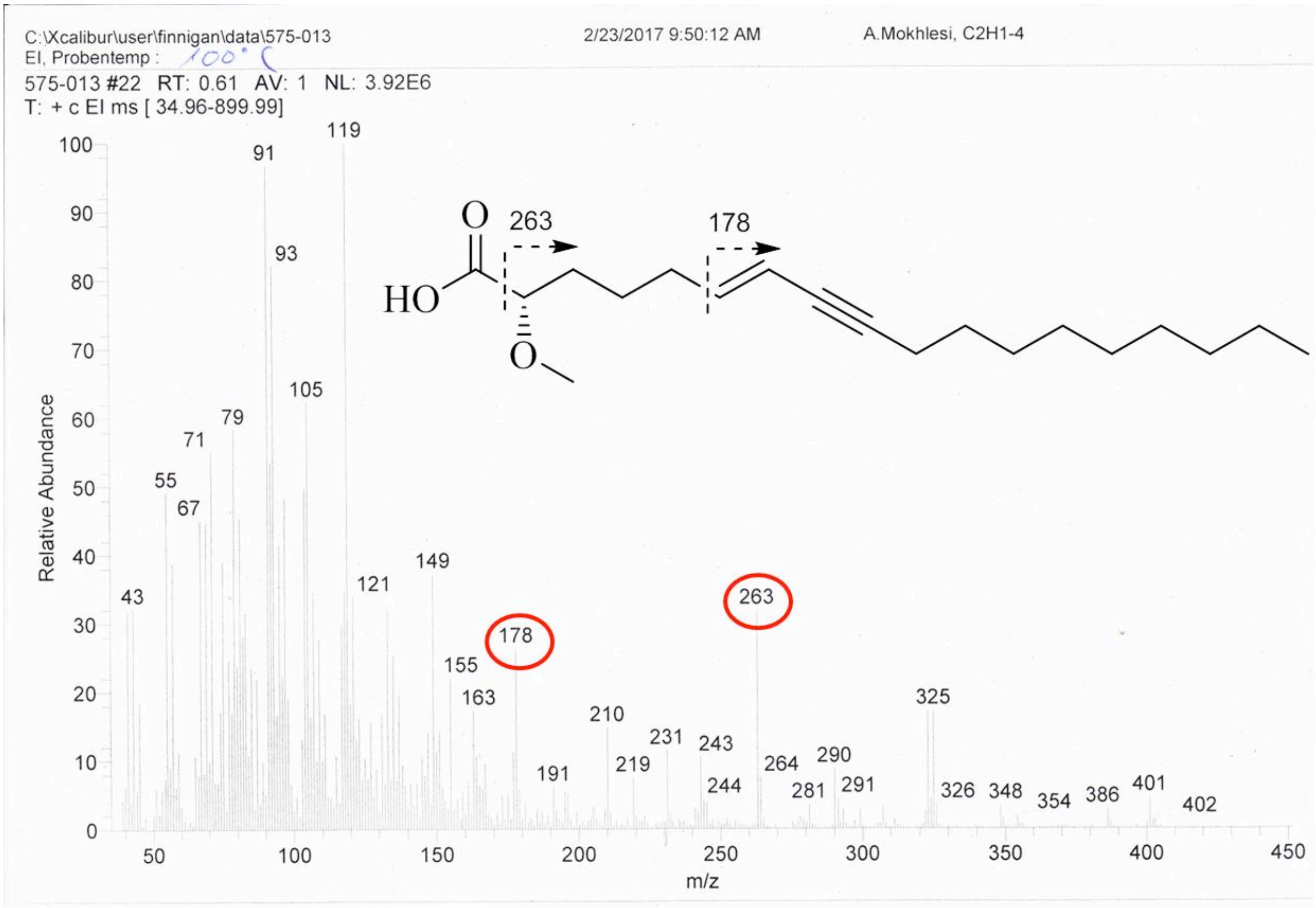

S1-5. EIMS spectrum of **1**

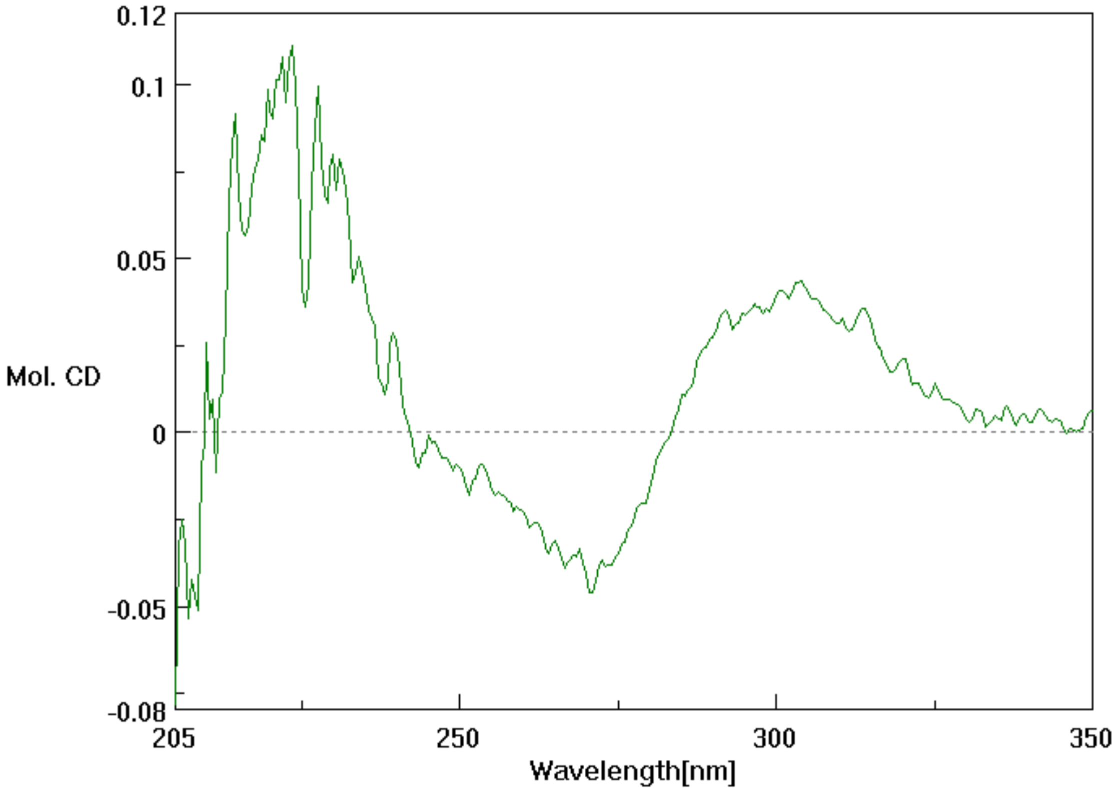

S1-6. CD spectrum of **1**

0.3 mg in 1.5 mL MeOH
c = 6.4838E-4 M
cell length: 1 cm
accumulation: 5
sensitivity: high (5 mdeg)

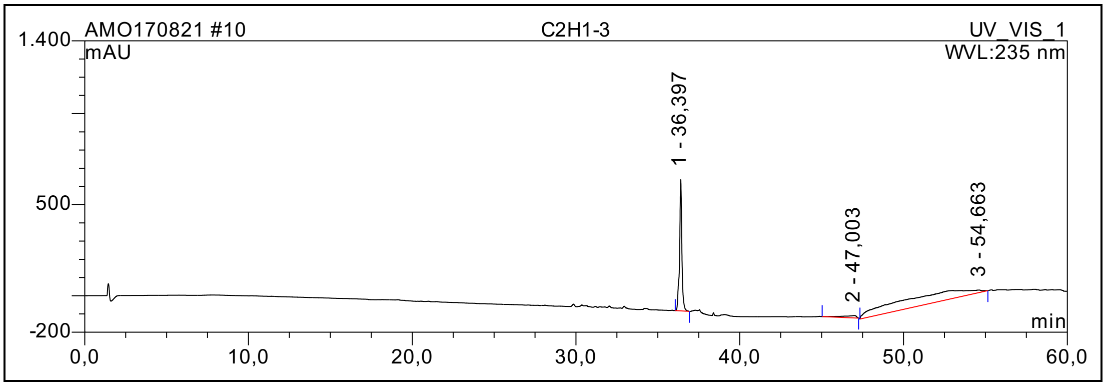

S1-7. HPLC chromatogram of **1**

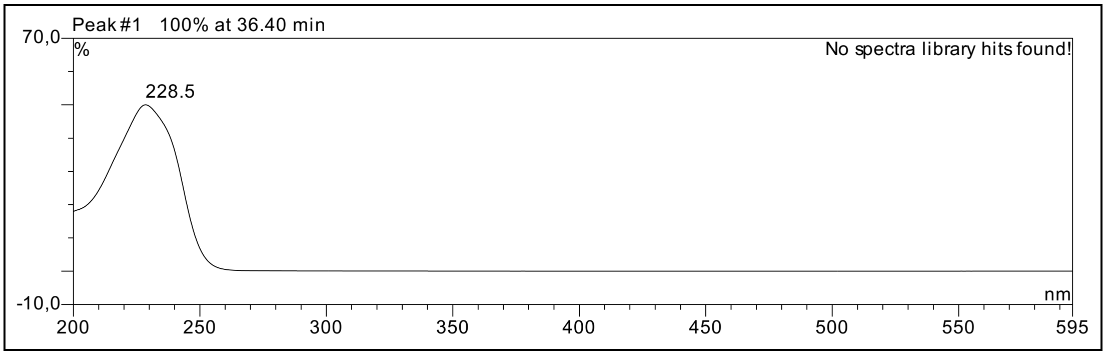

S1-8. UV spectrum of **1**

S2. Cinachylenic acid C (2)

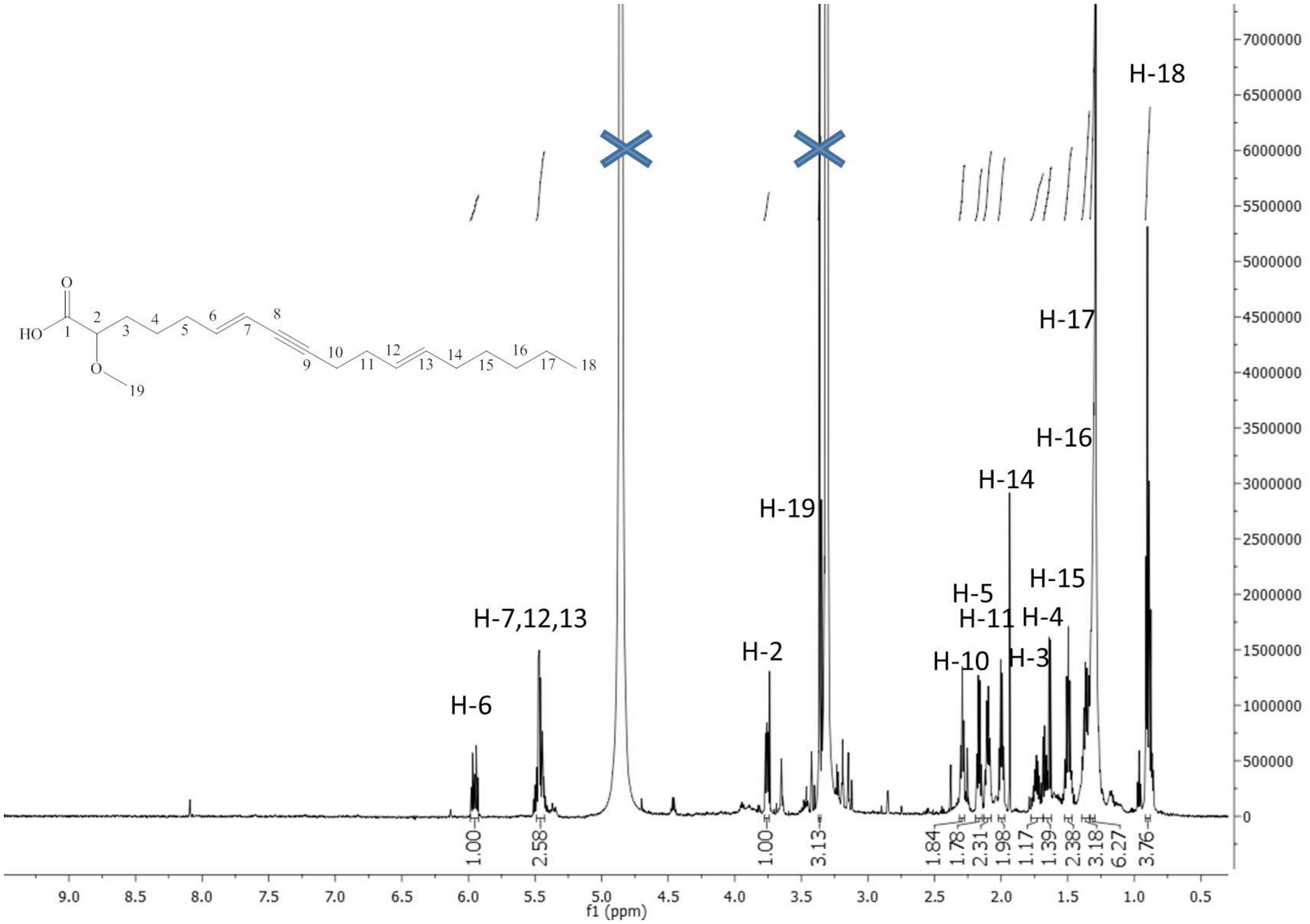

S2-1. ^{1}H NMR (MeOH-d_4, 600 MHz) spectrum of **2**

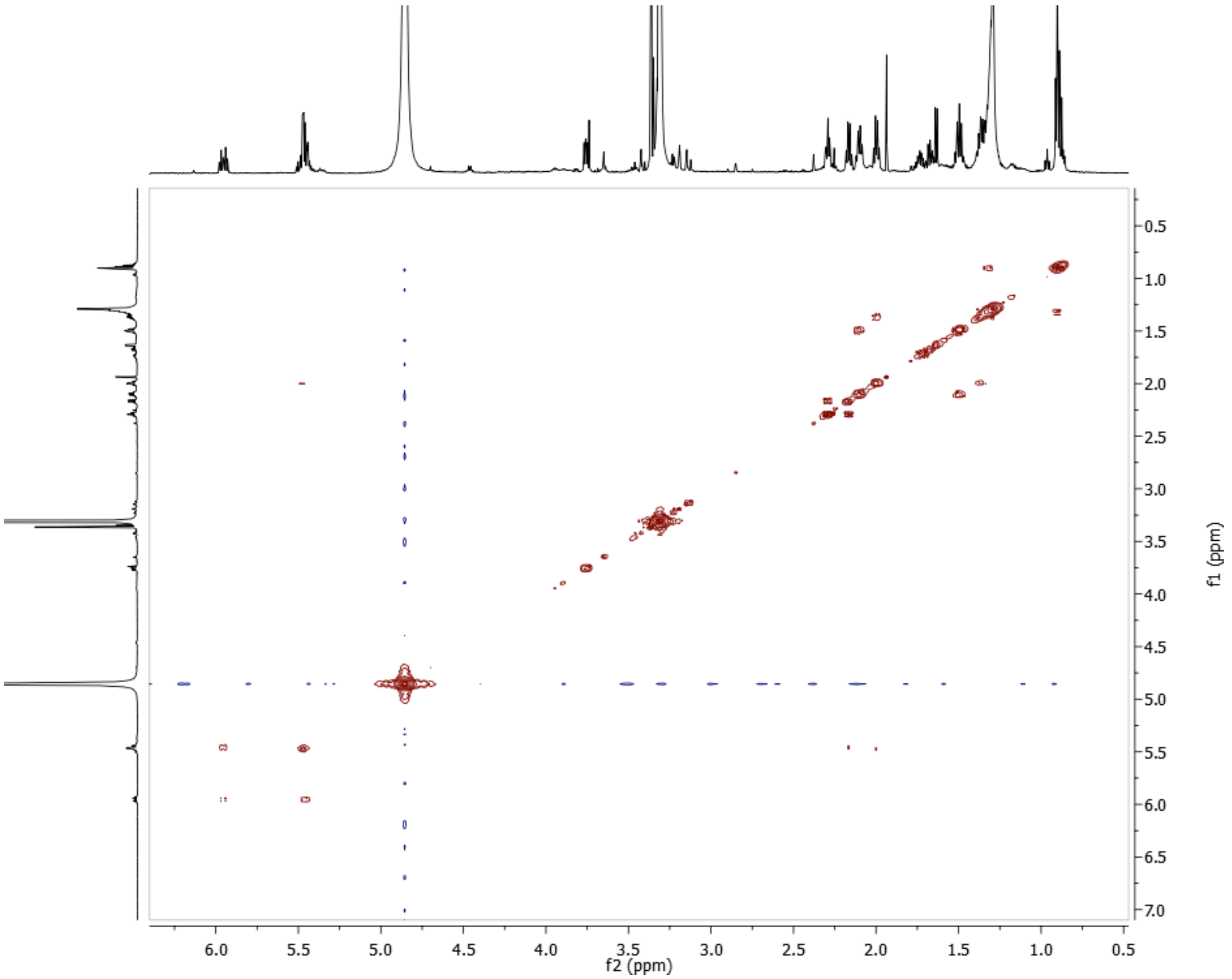

S2-2. COSY NMR (MeOH-*d*$_4$, 600 MHz) spectrum of **2**

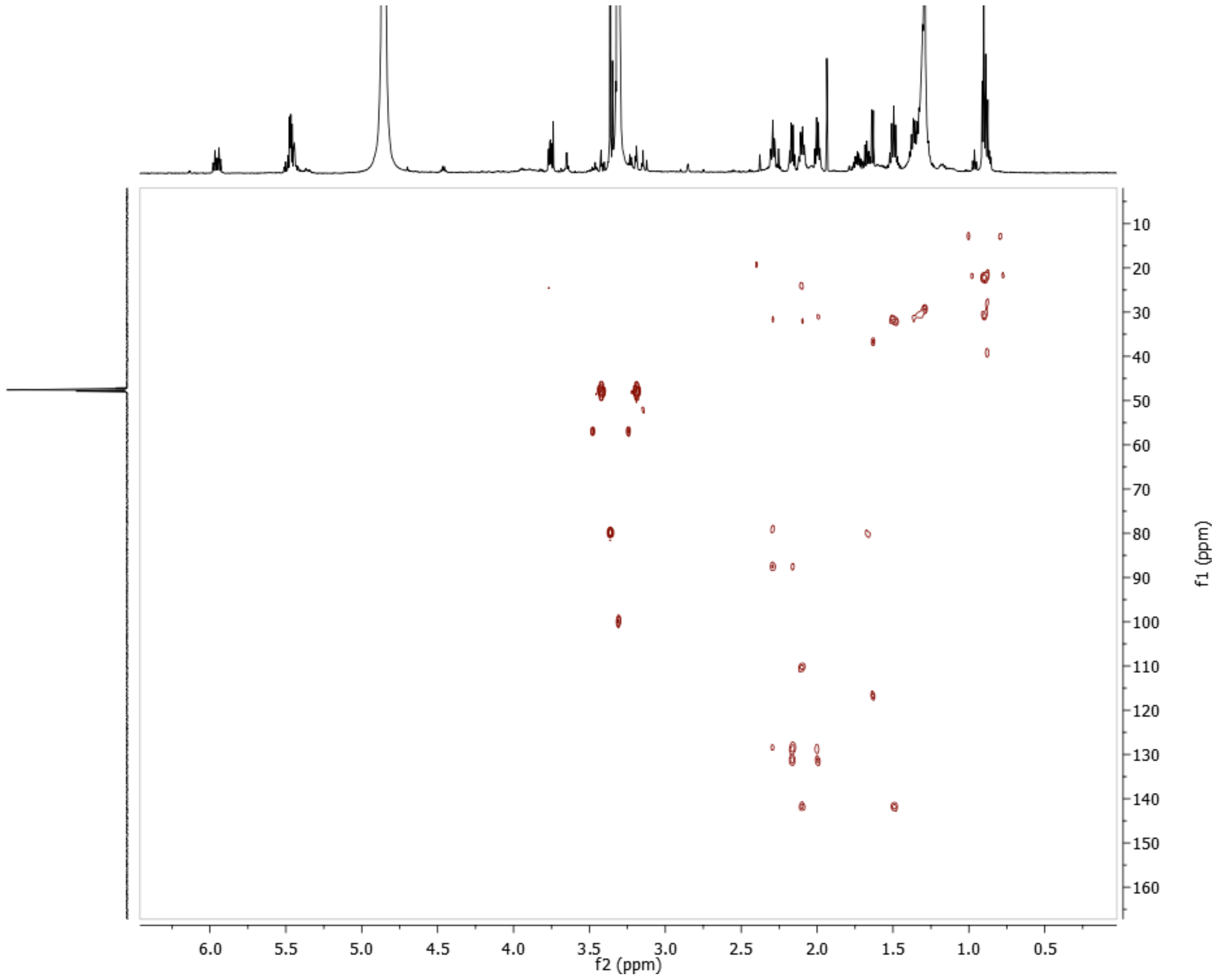

S2-3. HMBC NMR (MeOH-d_4, 600 MHz) spectrum of **2**

Mass Spectrum SmartFormula Report

Analysis Info

Analysis Name	D:\Data\spektren2016\Proksch16HR000173.d	Acquisition Date	7/7/2016 9:38:36 AM
Method	tune_low_new.m	Operator	Peter Tommes
Sample Name	Amin Mokhlesi C2 E3-3-2 (CH3OH)	Instrument	maXis 288882.20213
Comment			

Acquisition Parameter

Source Type	ESI	Ion Polarity	Positive	Set Nebulizer	0.3 Bar
Focus	Not active	Set Capillary	4000 V	Set Dry Heater	180 °C
Scan Begin	50 m/z	Set End Plate Offset	-500 V	Set Dry Gas	4.0 l/min
Scan End	1500 m/z	Set Collision Cell RF	600.0 Vpp	Set Divert Valve	Source

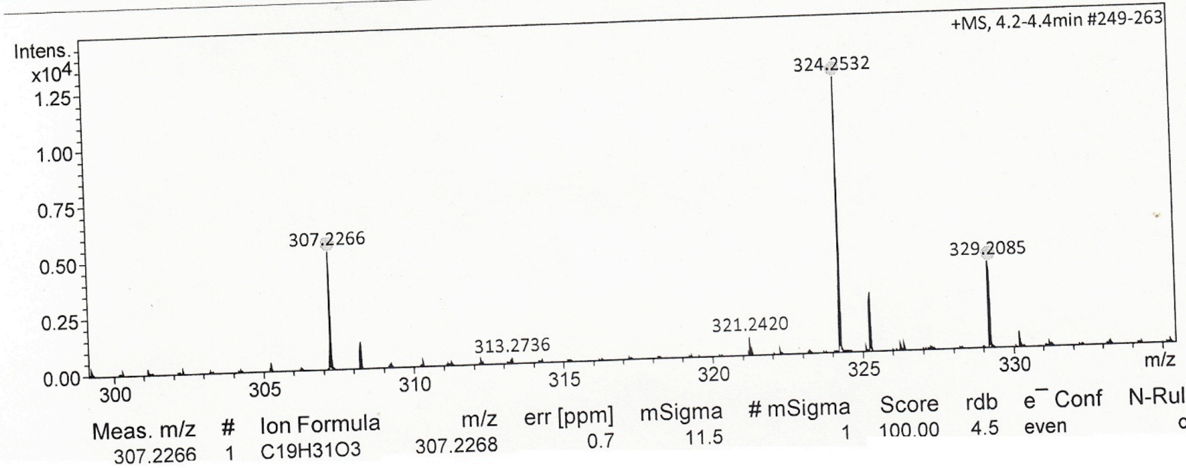

Meas. m/z	#	Ion Formula	m/z	err [ppm]	mSigma	# mSigma	Score	rdb	e⁻ Conf	N-Rule
307.2266	1	C19H31O3	307.2268	0.7	11.5	1	100.00	4.5	even	ok

S2-4. HRESIMS spectrum of **2**

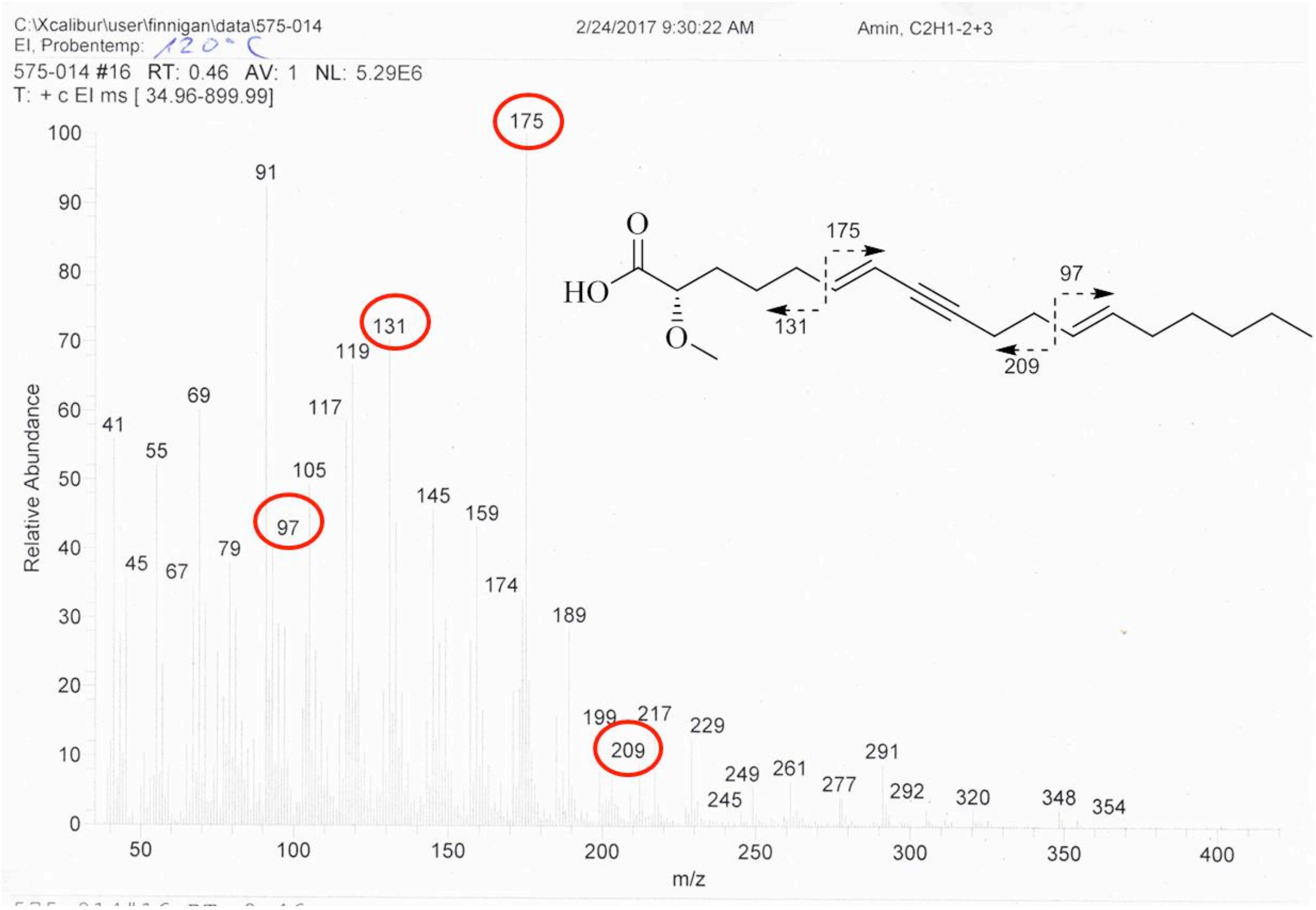

S2-5. EIMS spectrum of **2**

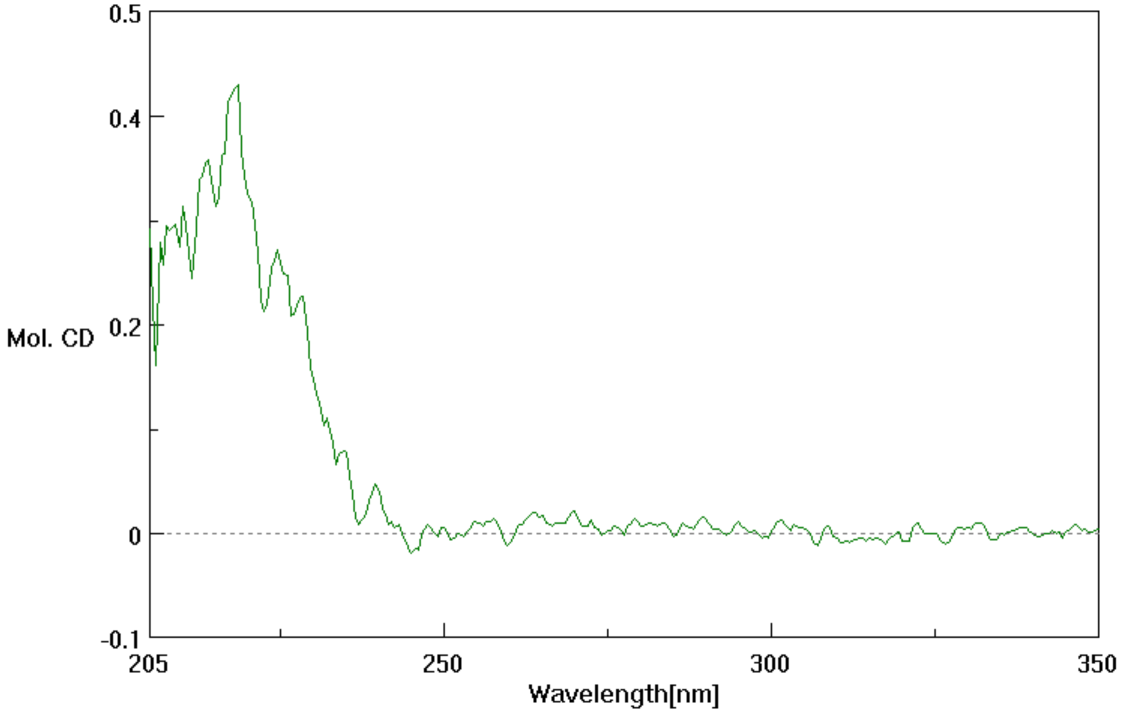

S2-6. CD spectrum of **2**

0.3 mg in 2.5 mL MeOH

c = 3.9159E-4 M

cell length: 1 cm

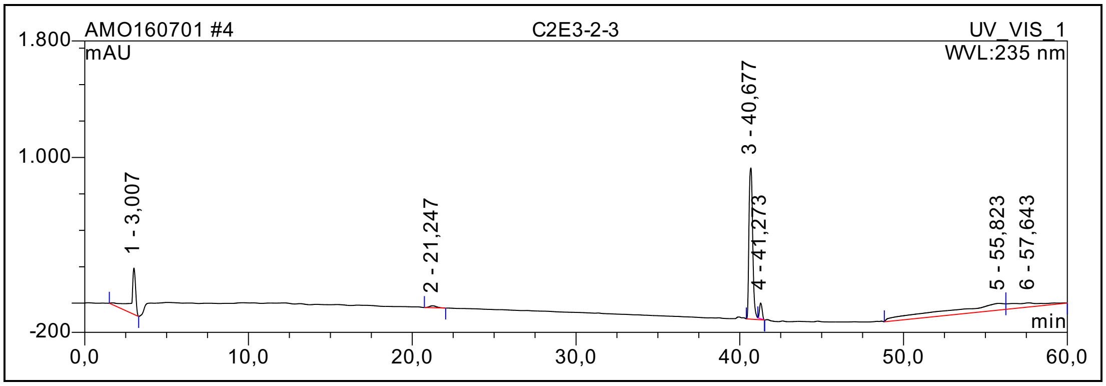

S2-7. HPLC chromatogram of **2**

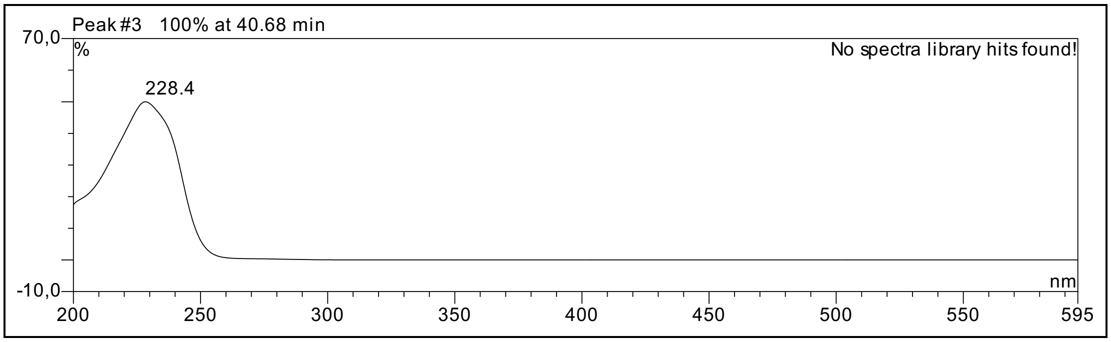

S2-8. UV spectrum of **2**

S3. Cinachylenic acid D (3)

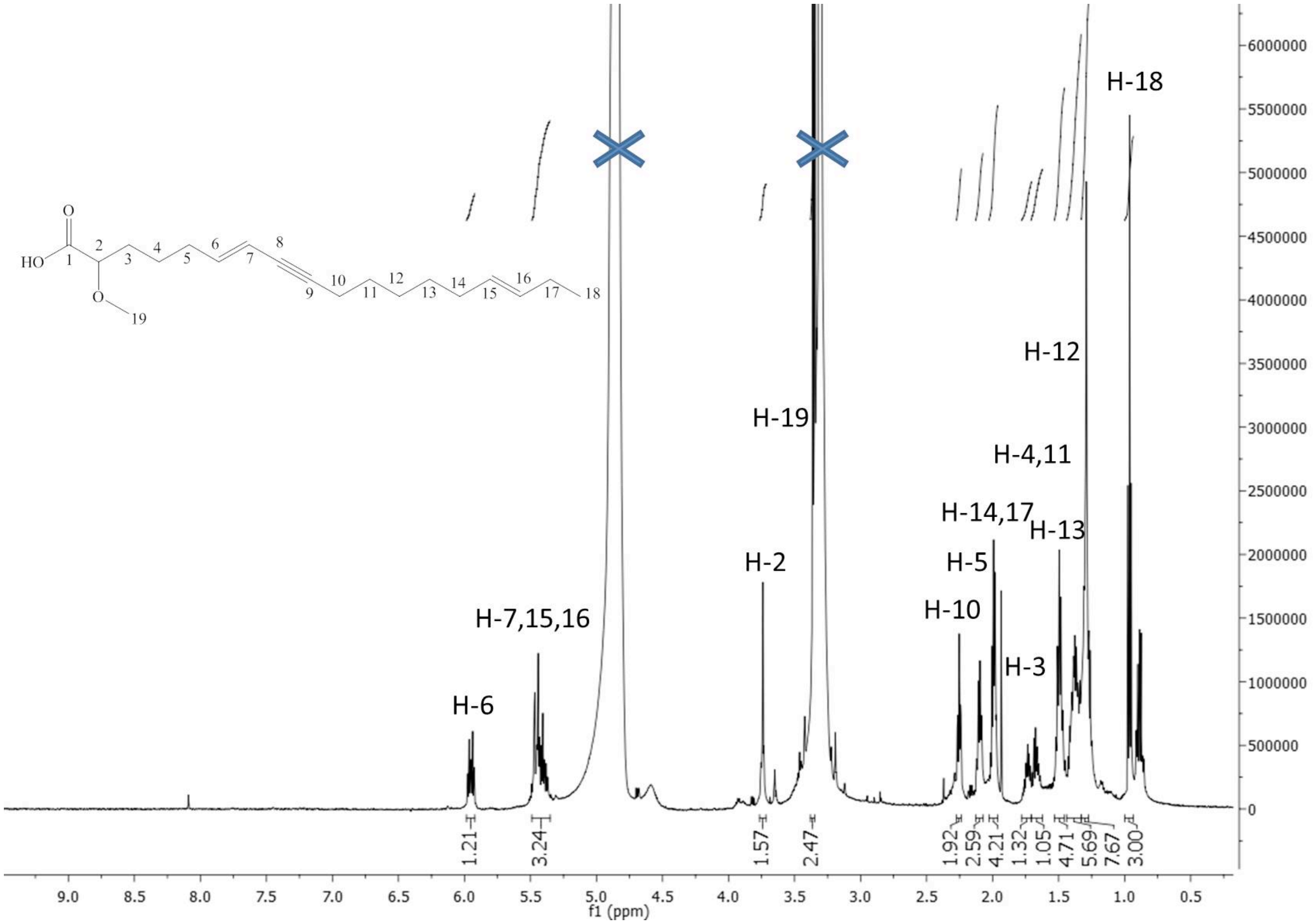

S3-1. ^{1}H NMR (MeOH-d_4, 600 MHz) spectrum of **3**

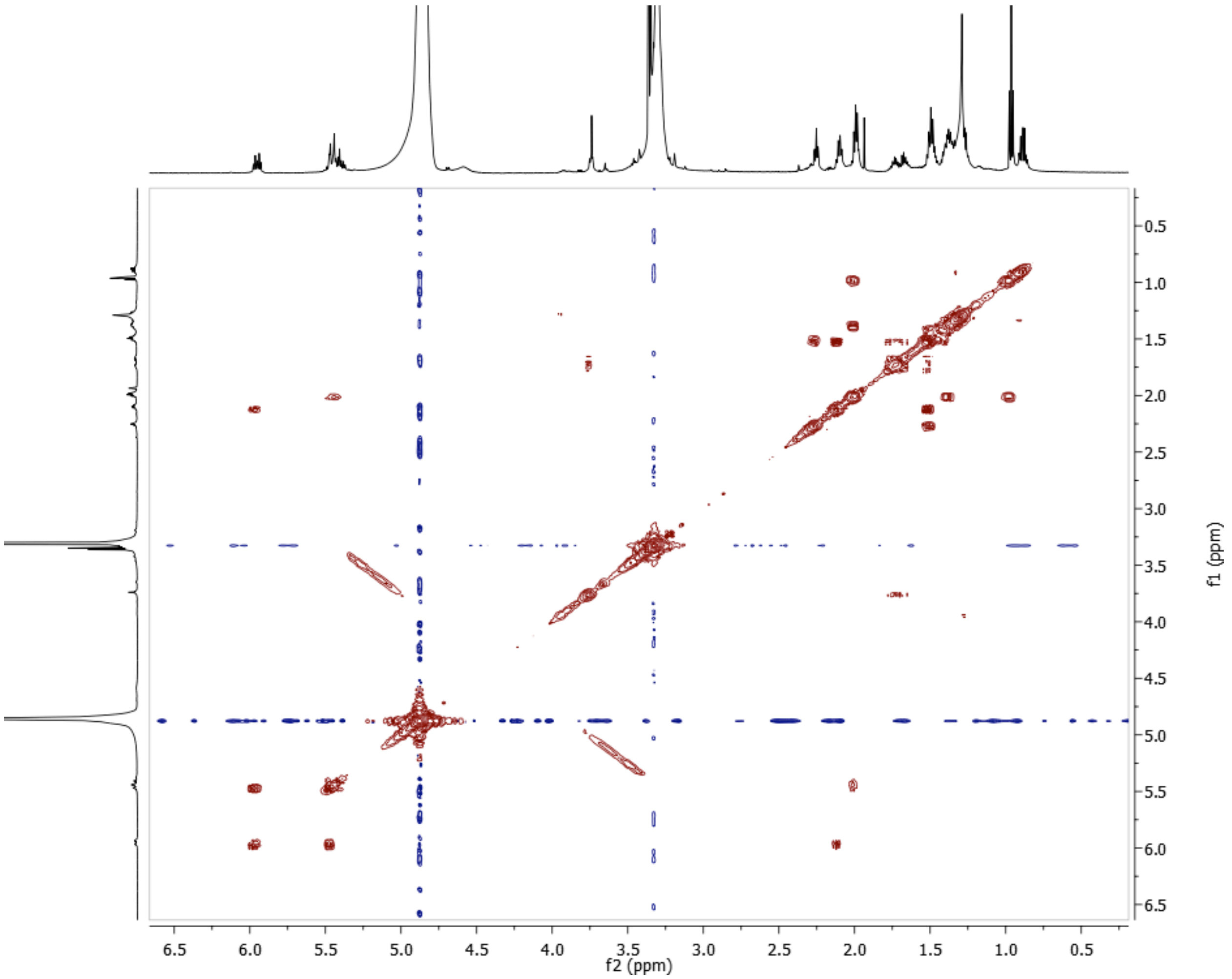

S3-2. COSY NMR (MeOH-d_4, 600 MHz) spectrum of **3**

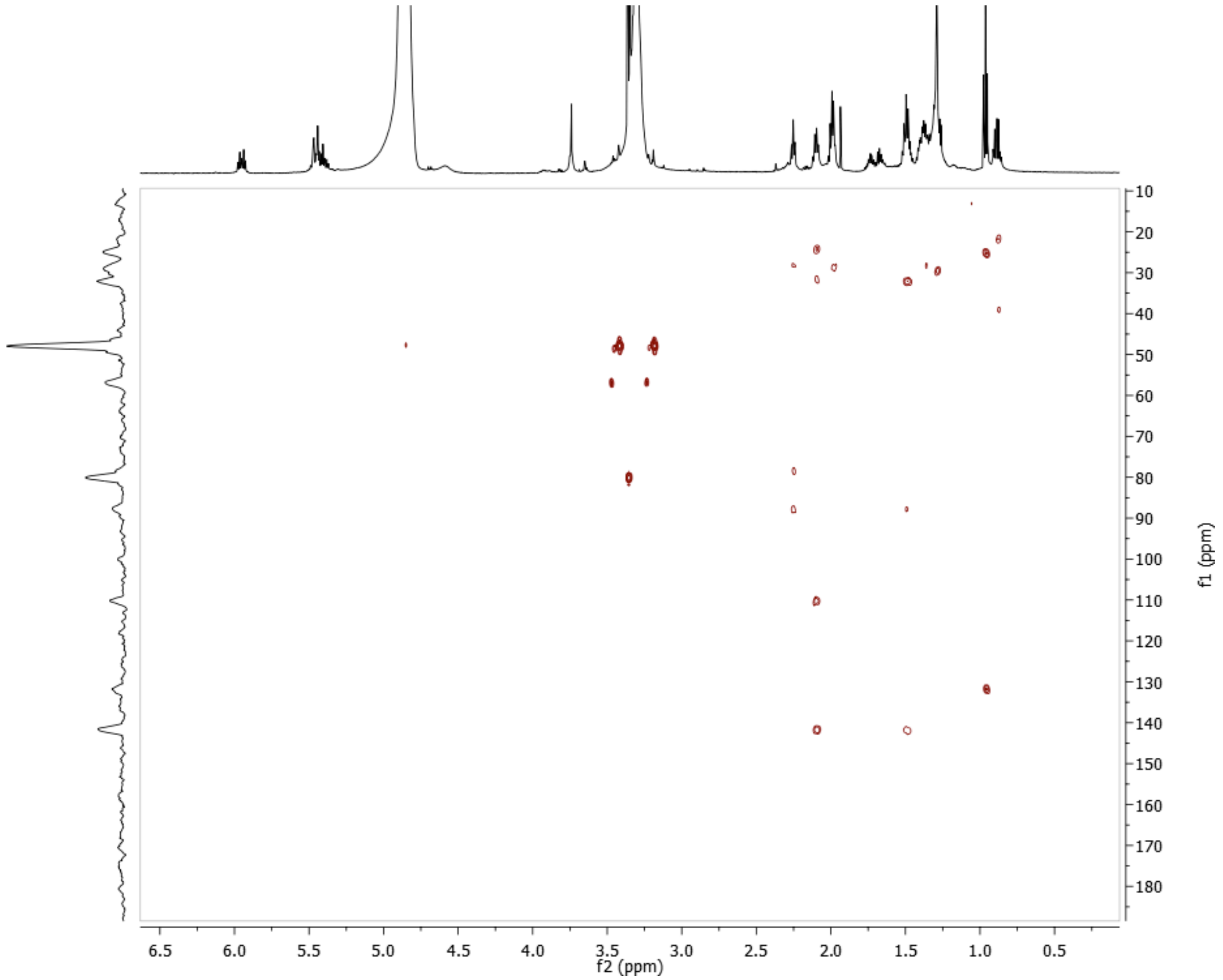

S3-3. HMBC NMR (MeOH-d_4, 600 MHz) spectrum of **3**

Mass Spectrum SmartFormula Report

Analysis Info

Analysis Name	D:\Data\spektren 2017\Proksch17HR000112.d	Acquisition Date	3/17/2017 12:35:16 PM
Method	tune_low_new.m	Operator	Peter Tommes
Sample Name	Amin C2E3-2-3 (CH3OH)	Instrument	maXis 288882.20213
Comment			

Acquisition Parameter

Source Type	ESI	Ion Polarity	Positive	Set Nebulizer	0.3 Bar
Focus	Not active	Set Capillary	4000 V	Set Dry Heater	180 °C
Scan Begin	50 m/z	Set End Plate Offset	-500 V	Set Dry Gas	4.0 l/min
Scan End	1500 m/z	Set Collision Cell RF	600.0 Vpp	Set Divert Valve	Source

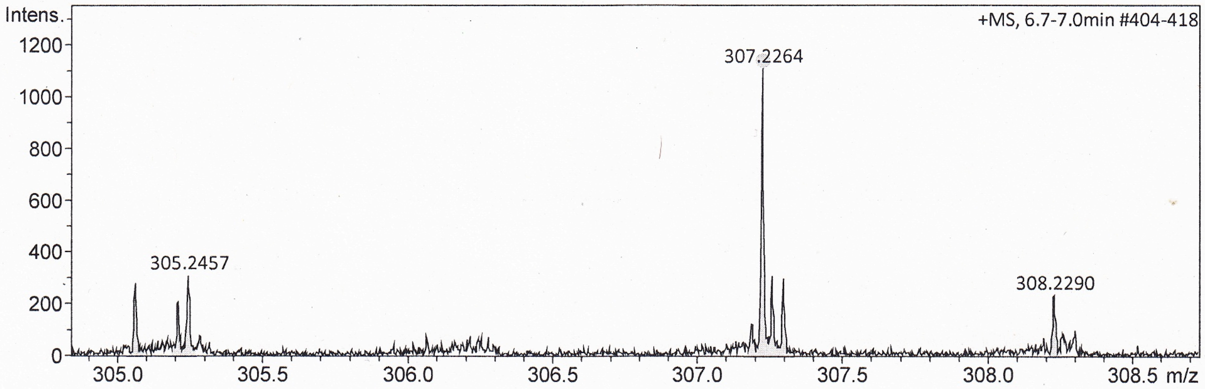

Meas. m/z	#	Ion Formula	m/z	err [ppm]	mSigma	# mSigma	Score	rdb	e⁻ Conf	N-Rule
307.2264	1	C19H31O3	307.2268	1.2	69.9	1	100.00	4.5	even	ok

S3-4. HRESIMS spectrum of **3**

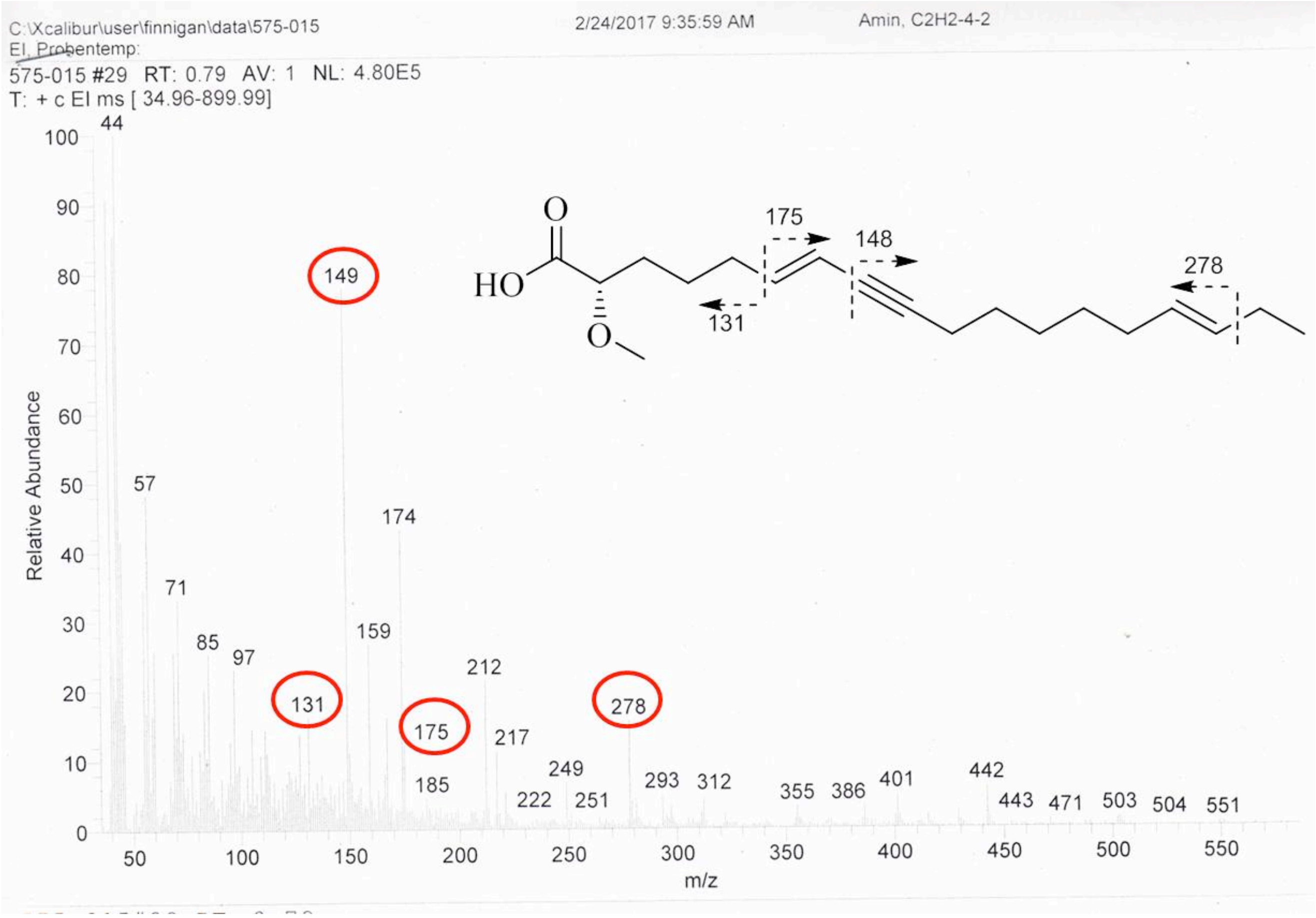

S3-5. EIMS spectrum of **3**

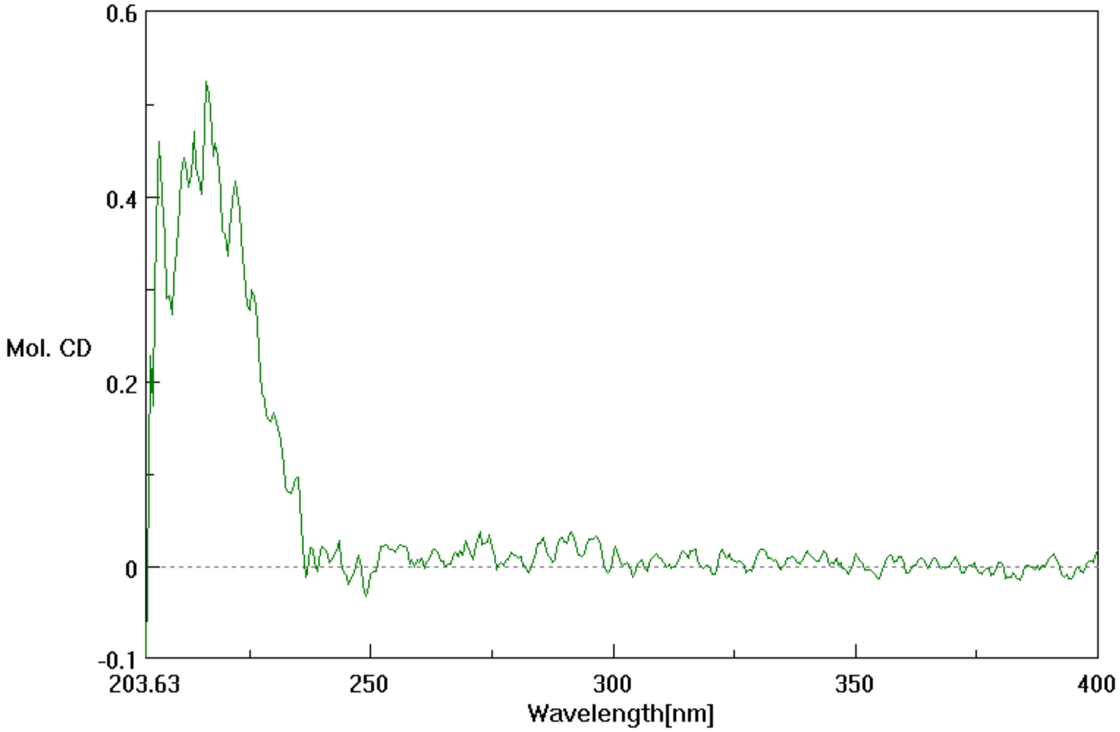

S3-6. CD spectrum of **3**

0.3 mg in 4 mL MeOH

c = 2.4475E-4 M

cell length: 1 cm

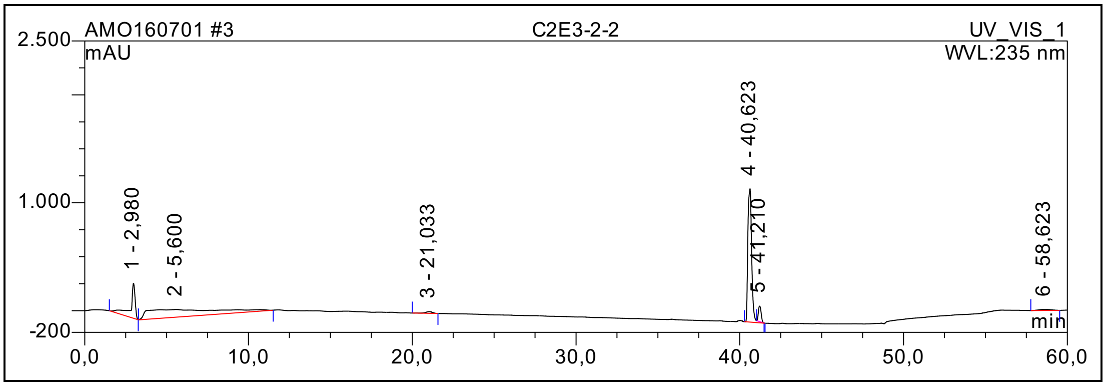

S3-7. HPLC chromatogram of **3**

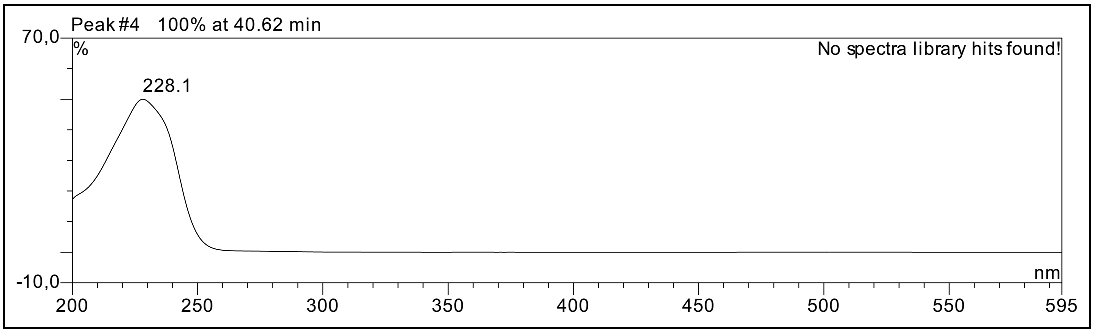

S3-8. UV spectrum of **3**

S4. Cinachylenic acid A (4)

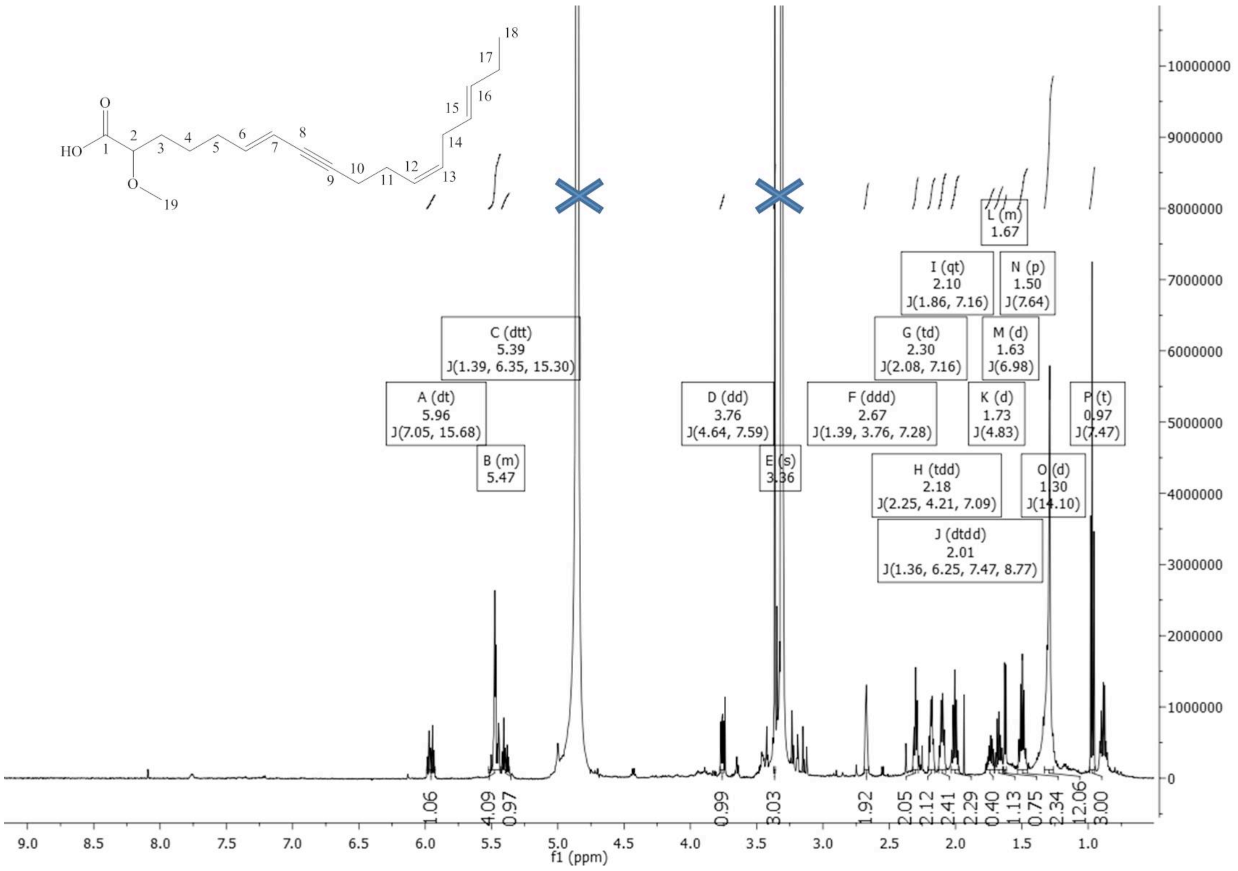

S4-1. ^{1}H NMR (MeOH-d_4, 600 MHz) spectrum of **4**

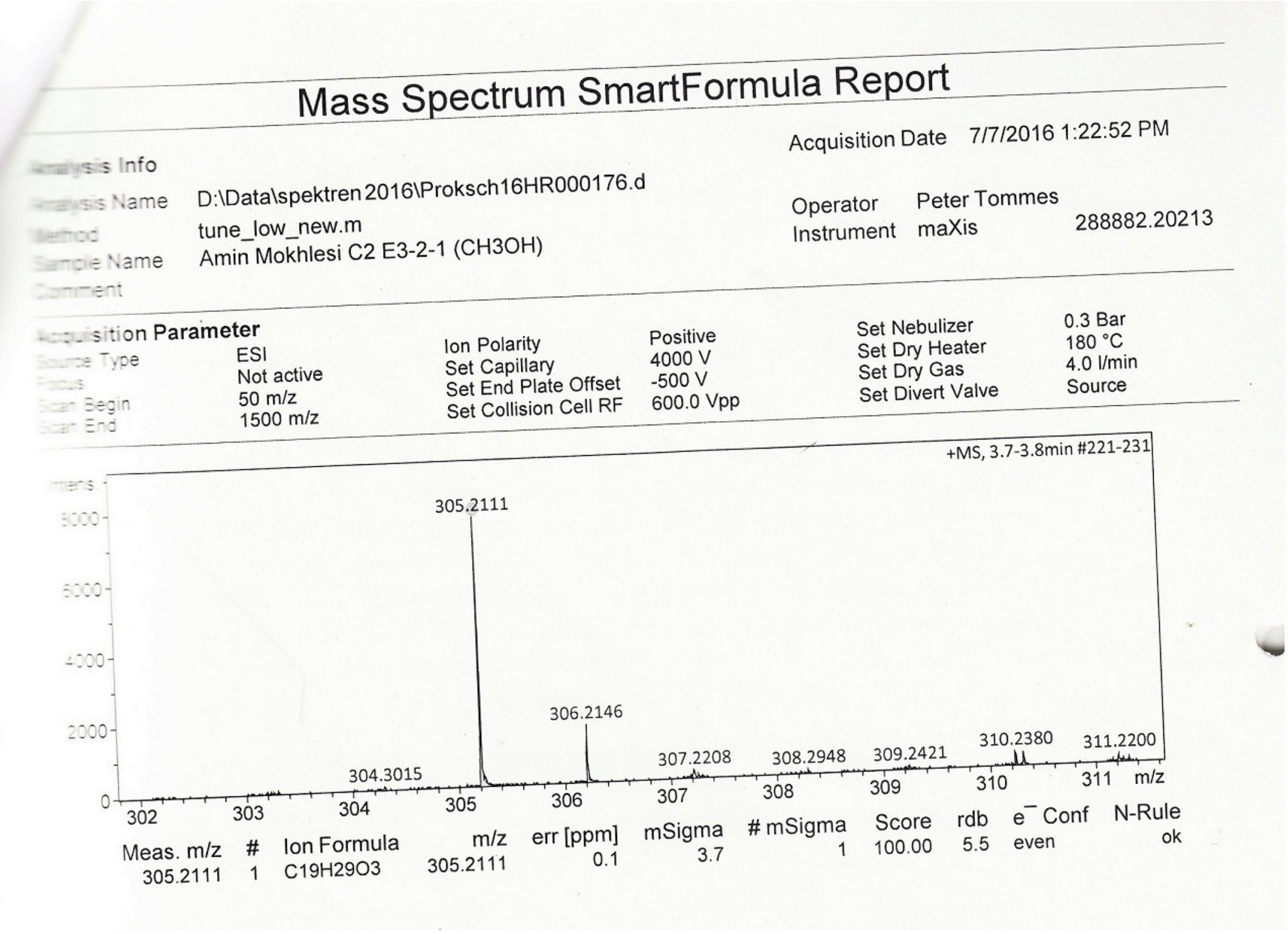

S4-2. HRESIMS spectrum of **4**

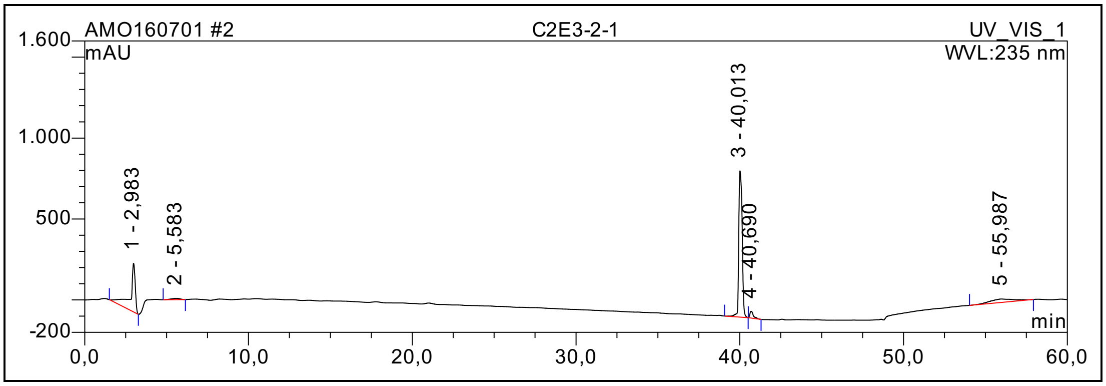

S4-3. HPLC chromatogram of **4**

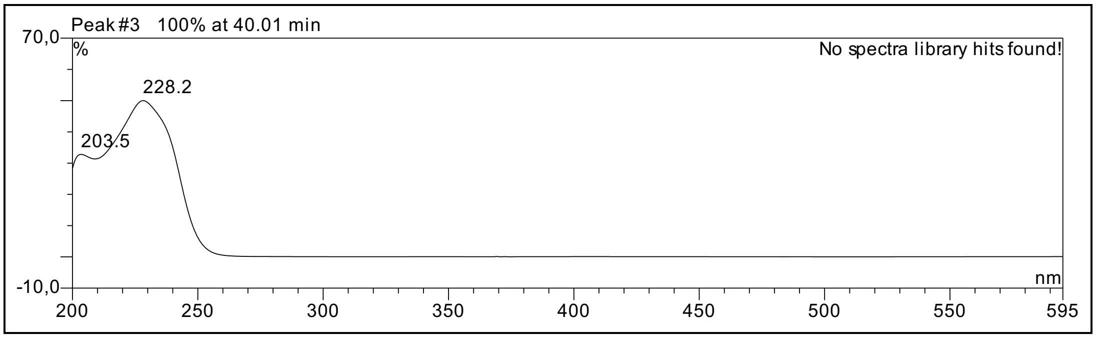

S4-4. UV spectrum of **4**

S5. Cinachyrazole A (5)

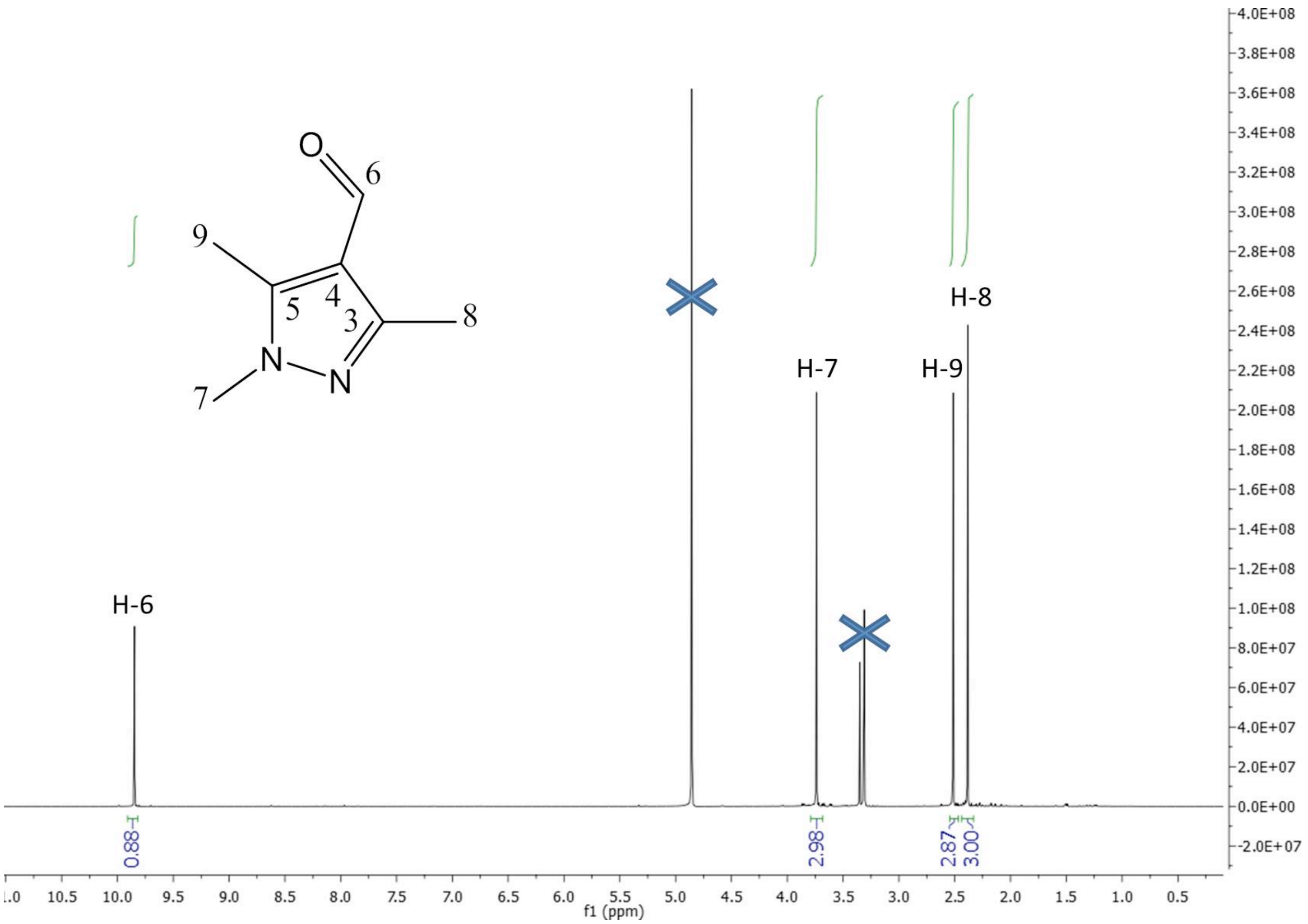

S5-1. ^{1}H NMR (MeOH-*d*$_4$, 600 MHz) spectrum of **5**

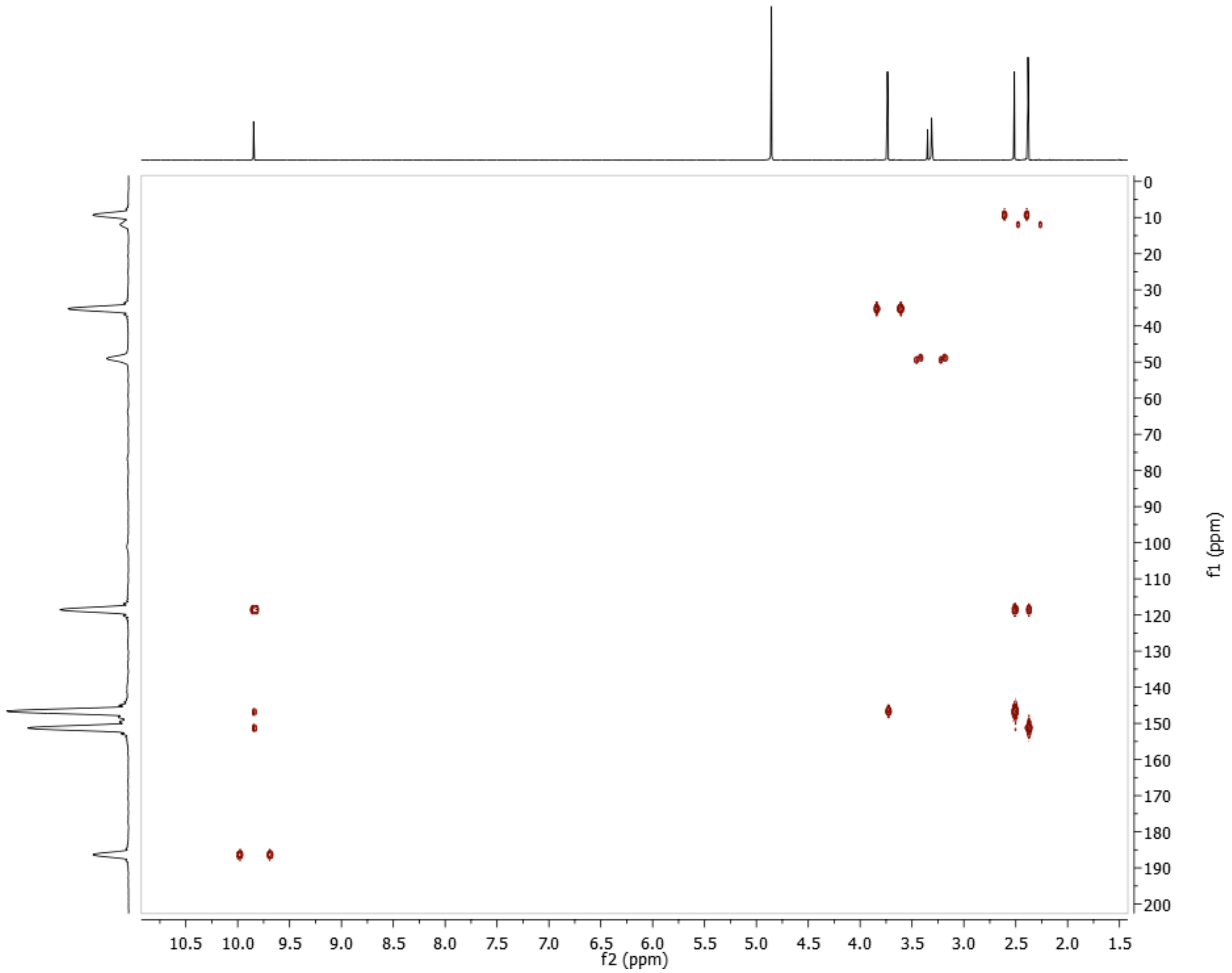

S5-2. HMBC NMR (MeOH-*d*$_4$, 600 MHz) spectrum of **5**

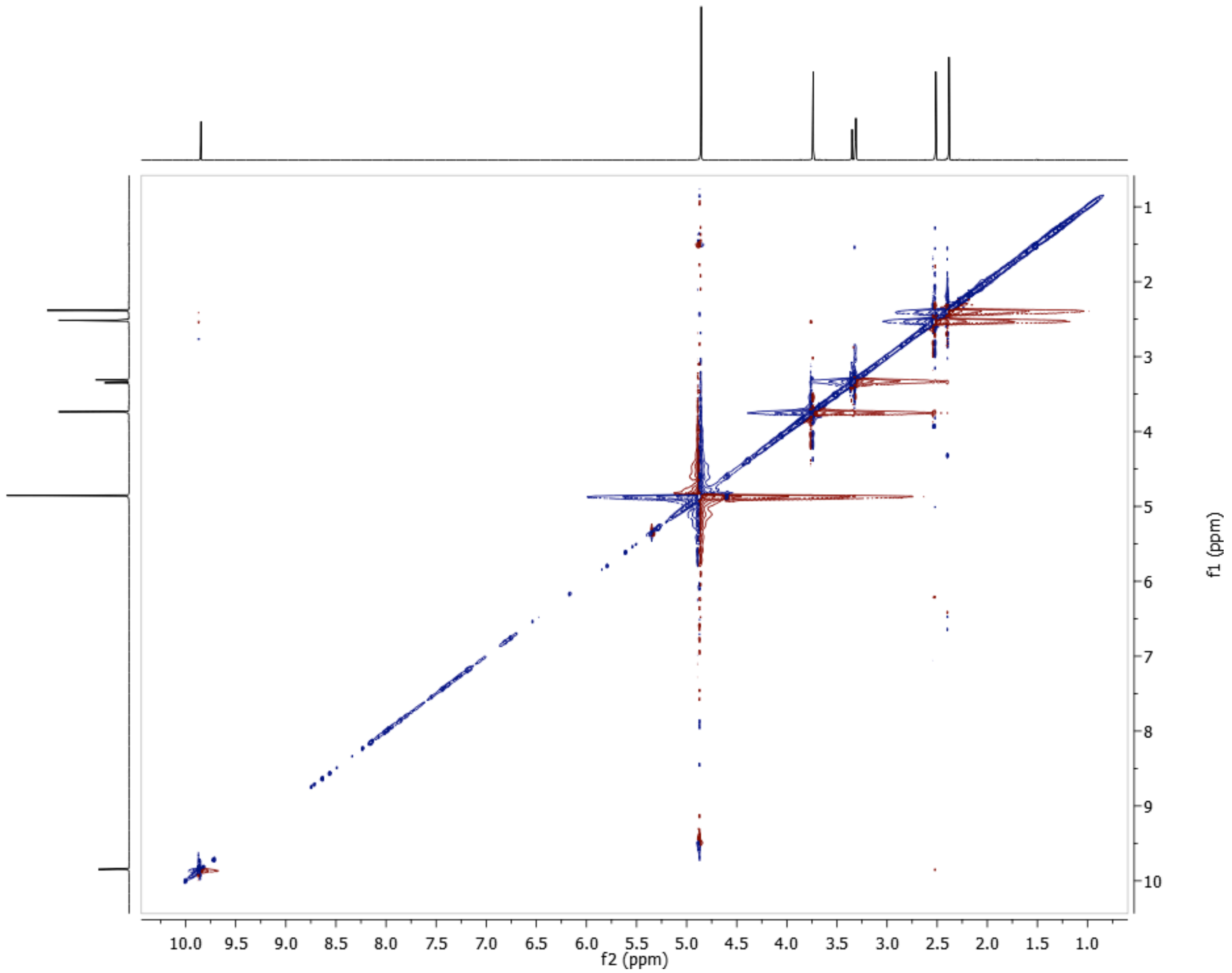

S5-3. ROESY NMR (MeOH-*d*4, 600 MHz) spectrum of **5**

Mass Spectrum SmartFormula Report

Analysis Info

Analysis Name	D:\Data\spektren 2017\Proksch17HR000008.d	Acquisition Date	1/9/2017 3:42:53 PM
Method	tune_low_new.m	Operator	Peter Tommes
Sample Name	Amin Mokhlesi C2E5-2 (CH3OH)	Instrument	maXis 288882.20213
Comment			

Acquisition Parameter

Source Type	ESI	Ion Polarity	Positive	Set Nebulizer	0.3 Bar
Focus	Not active	Set Capillary	4000 V	Set Dry Heater	180 °C
Scan Begin	50 m/z	Set End Plate Offset	-500 V	Set Dry Gas	4.0 l/min
Scan End	1500 m/z	Set Collision Cell RF	600.0 Vpp	Set Divert Valve	Source

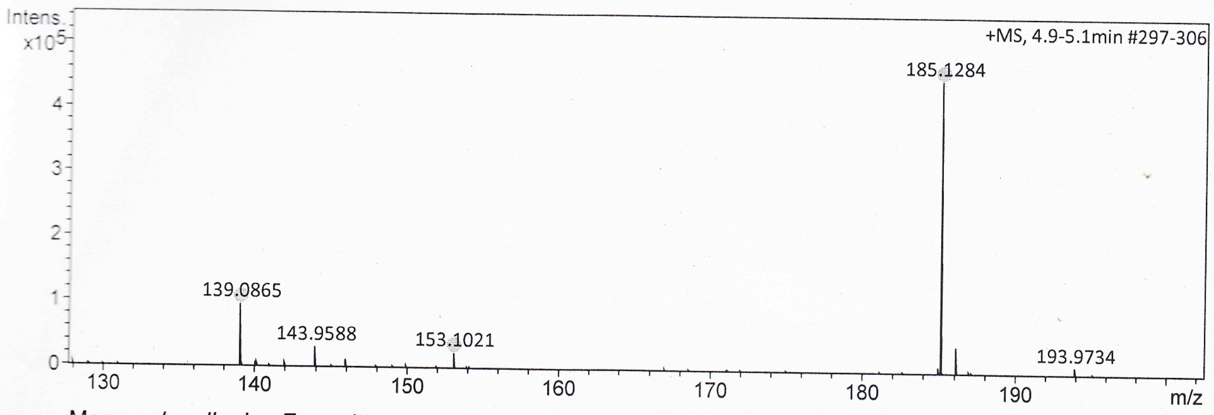

Meas. m/z	#	Ion Formula	m/z	err [ppm]	mSigma	# mSigma	Score	rdb	e⁻ Conf	N-Rule
139.0865	1	C7H11N2O	139.0866	0.8	8.2	1	100.00	3.5	even	ok
153.1021	1	C8H13N2O	153.1022	1.0	3.8	1	100.00	3.5	even	ok
185.1284	1	C9H17N2O2	185.1285	0.0	10.3	1	100.00	2.5	even	ok

S5-4. HRESIMS spectrum of **5**

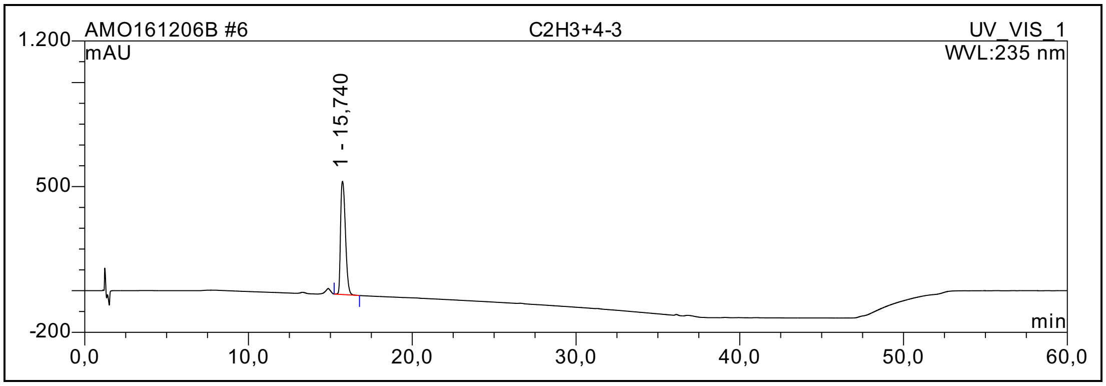

S5-5. HPLC chromatogram of **5**

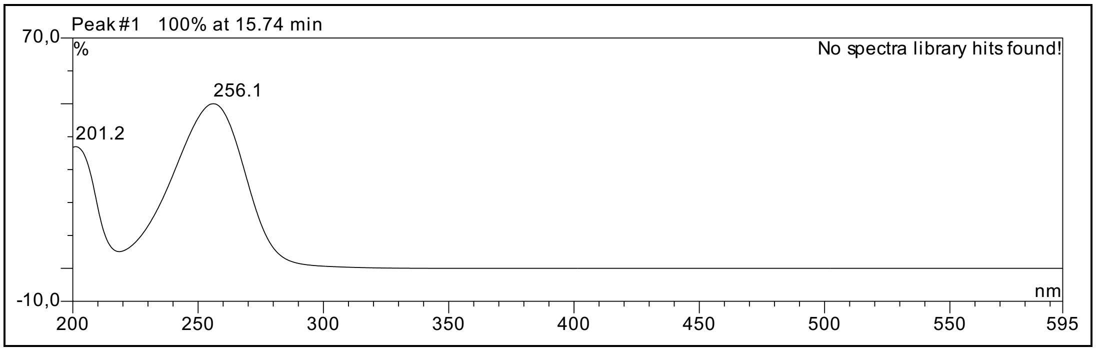

S5-6. UV spectrum of **5**

S5-7. Comparison of UV data of **5** with those reported in the literature

Compound	5[1]	5[2]
UV (λ_{max}, MeOH)	201; 256	206; 252

[1] Isolated in this study, [2] UV data of **5** reported in the literature: Mortikov, V.Y.; Rodinovskaya, L.A.; Fedorov, A.E.; Shestopalov, A.M.; Belyakov, P.A. Synthesis of heterocyclic compounds from 4-formylpyrazoles. *Russian Chemical Bulletin* **2014**, *63*, 443-456. DOI: 10.1007/s11172-014-0451-8.

S5-8. Comparison of ^{1}H NMR (600 MHz) data of **5** with those reported in the literature

**Position**	**5**[1] (MeOH-d_4, δ in ppm) ^{1}H (J in Hz)	**5**[2] (DMSO-d_6, δ in ppm) ^{1}H (J in Hz)
6	9.85, s	9.81, s
7	3.74, s	3.68, s
8	2.38, s	2.29, s
9	2.51, s	2.45, s

[1] Isolated in this study, [2] ^{1}H NMR data of **5** reported in the literature: Mortikov, V.Y.; Rodinovskaya, L.A.; Fedorov, A.E.; Shestopalov, A.M.; Belyakov, P.A. Synthesis of heterocyclic compounds from 4-formylpyrazoles. *Russian Chemical Bulletin* **2014**, *63*, 443-456. DOI: 10.1007/s11172-014-0451-8.

S6. Cinachyrazole B (6)

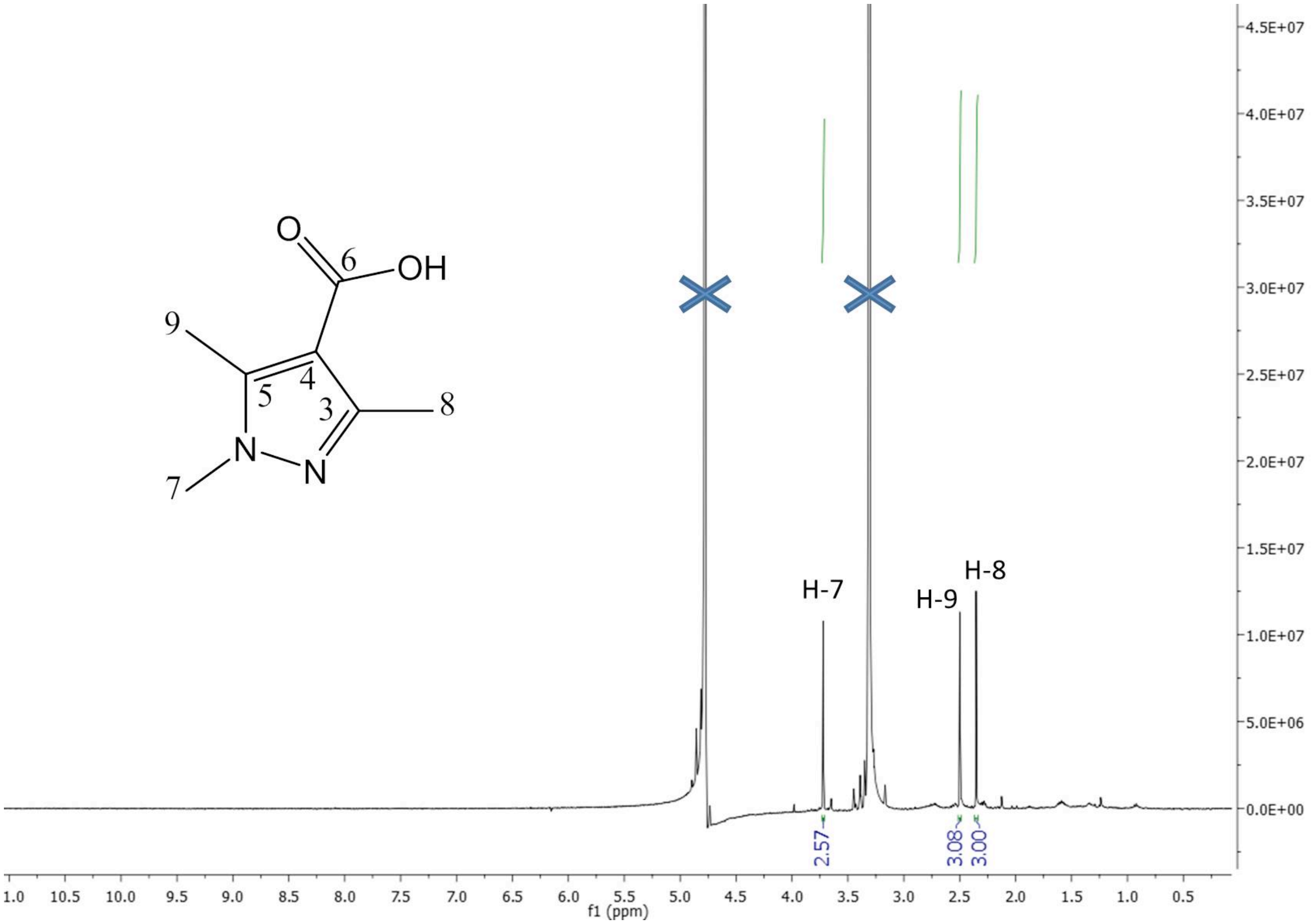

S6-1. ^{1}H NMR (MeOH-*d4*, 600 MHz) spectrum of **6**

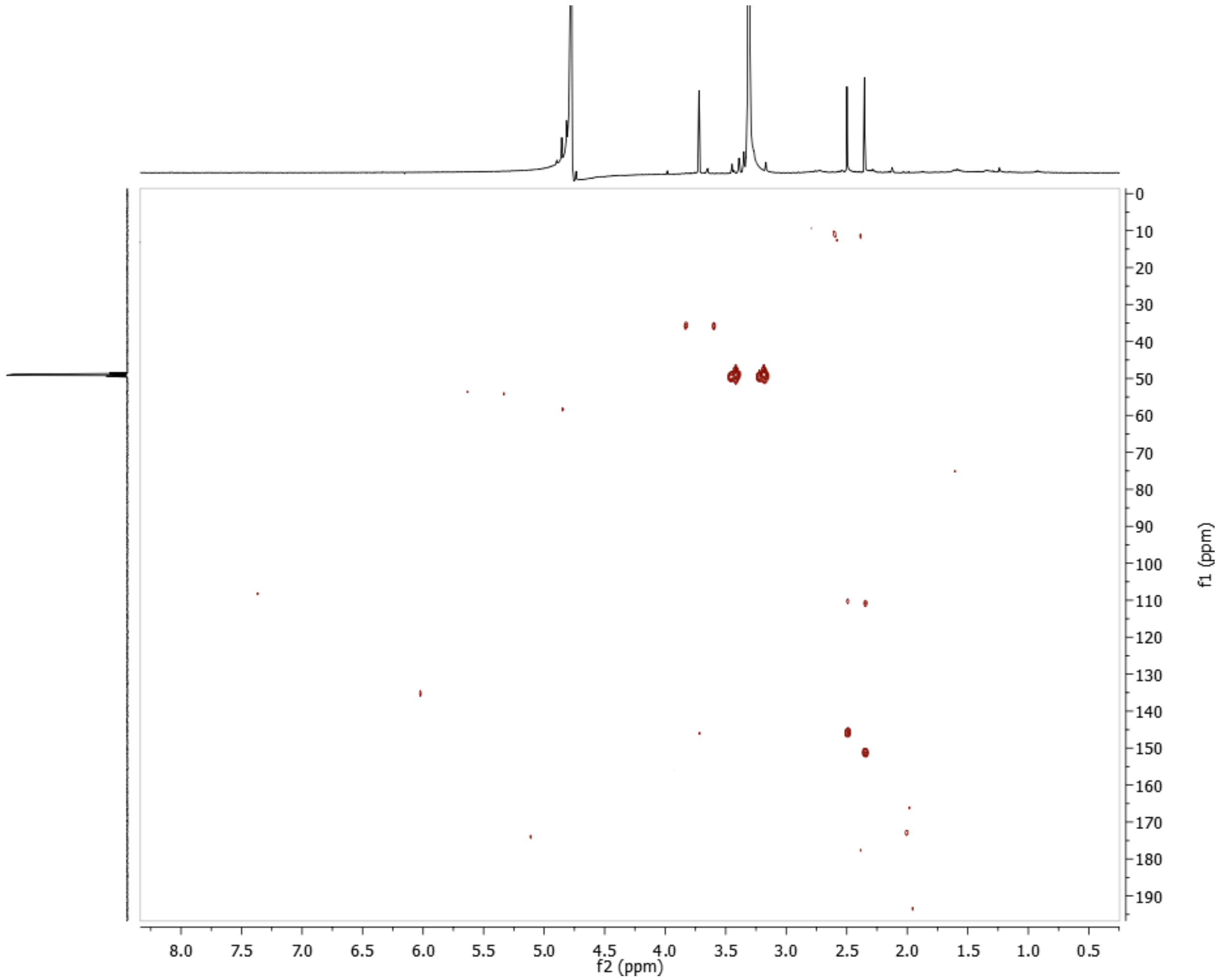

S6-2. HMBC NMR (MeOH-*d*$_4$, 600 MHz) spectrum of **6**

Mass Spectrum SmartFormula Report

Analysis Info		Acquisition Date	2/10/2017 9:29:17 AM
Analysis Name	D:\Data\spektren\2017\Proksch17HR000025.d		
Method	tune_low_new.m	Operator	Peter Tommes
Sample Name	Amin Mokhlesi C2B5-2 (CH3OH)	Instrument	maXis 288882.20213
Comment			

Acquisition Parameter					
Source Type	ESI	Ion Polarity	Positive	Set Nebulizer	0.3 Bar
Focus	Not active	Set Capillary	4000 V	Set Dry Heater	180 °C
Scan Begin	50 m/z	Set End Plate Offset	-500 V	Set Dry Gas	4.0 l/min
Scan End	1500 m/z	Set Collision Cell RF	600.0 Vpp	Set Divert Valve	Source

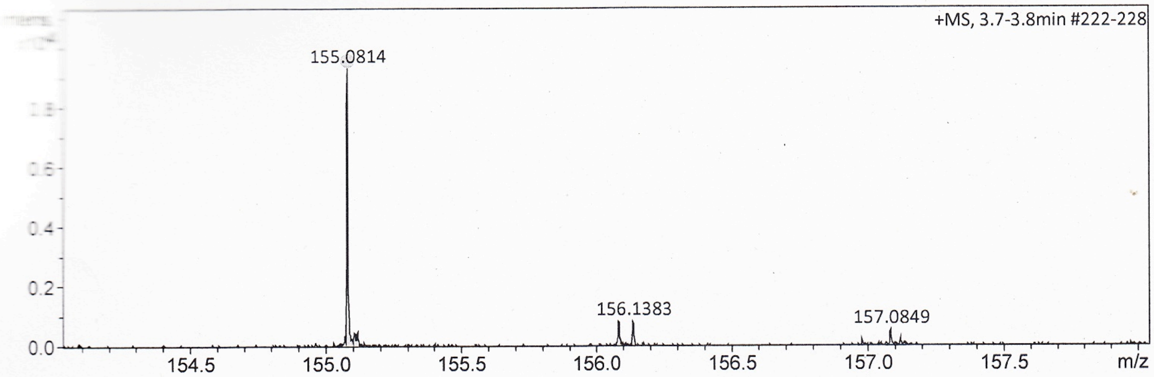

Meas. m/z	#	Ion Formula	m/z	err [ppm]	mSigma	# mSigma	Score	rdb	e⁻ Conf	N-Rule
155.0814	1	C7H11N2O2	155.0815	0.6	27.8	1	100.00	3.5	even	ok

S6-3. HRESIMS spectrum of **6**

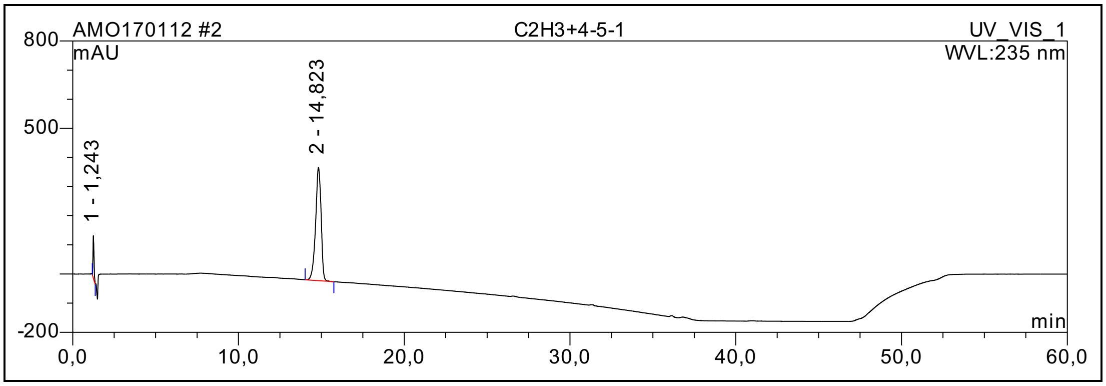

S6-4. HPLC chromatogram of **6**

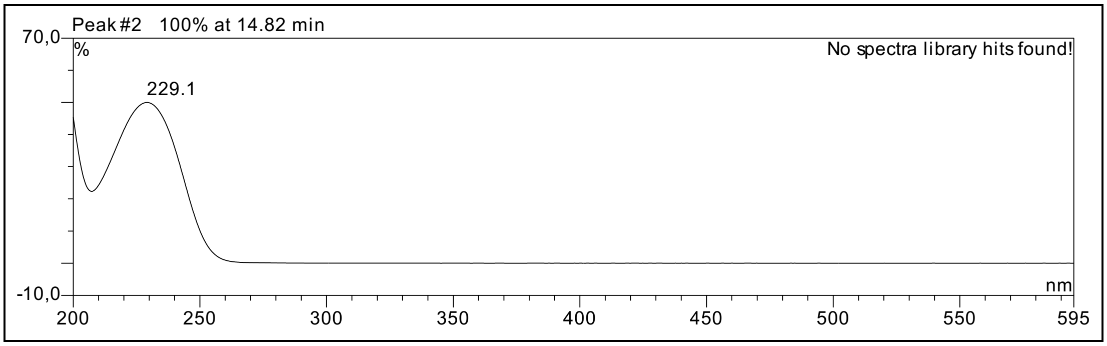

S6-5. UV spectrum of **6**

S6-6. Comparison of the ^{13}C NMR (150 MHz) data of **6** with those reported in the literature

Position	**6**[1] ^{13}C, type[2] (MeOH-d_4, δ in ppm)	**6**[4] ^{13}C, type (DMSO-d_6, δ in ppm)
3	151.2, C	148.7, C
4	110.5, C	109.1, C
5	145.7, C	143.5, C
6	– [3]	– [5]
7	35.8, CH_3	35.6, CH_3
8	– [3]	– [5]
9	11.5, CH_3	– [5]

[1] Isolated in this study, [2] Data extracted from HMBC spectra, [3] Not observed, [4] ^{13}C NMR data of **6** reported in the literature: Begtrup, M., Boyer, G., Cabildo, P., Cativiela, C., Claramunt, R.M., Elguero, J., García, J.I., Toiron, C., Vedsø, P. ^{13}C NMR of pyrazoles. *Magnetic resonance in chemistry* **1993**, *31*:107-68. DOI: 10.1002/mrc.1260310202, [5] Not reported.

S7. Cinachyrazole C (7)

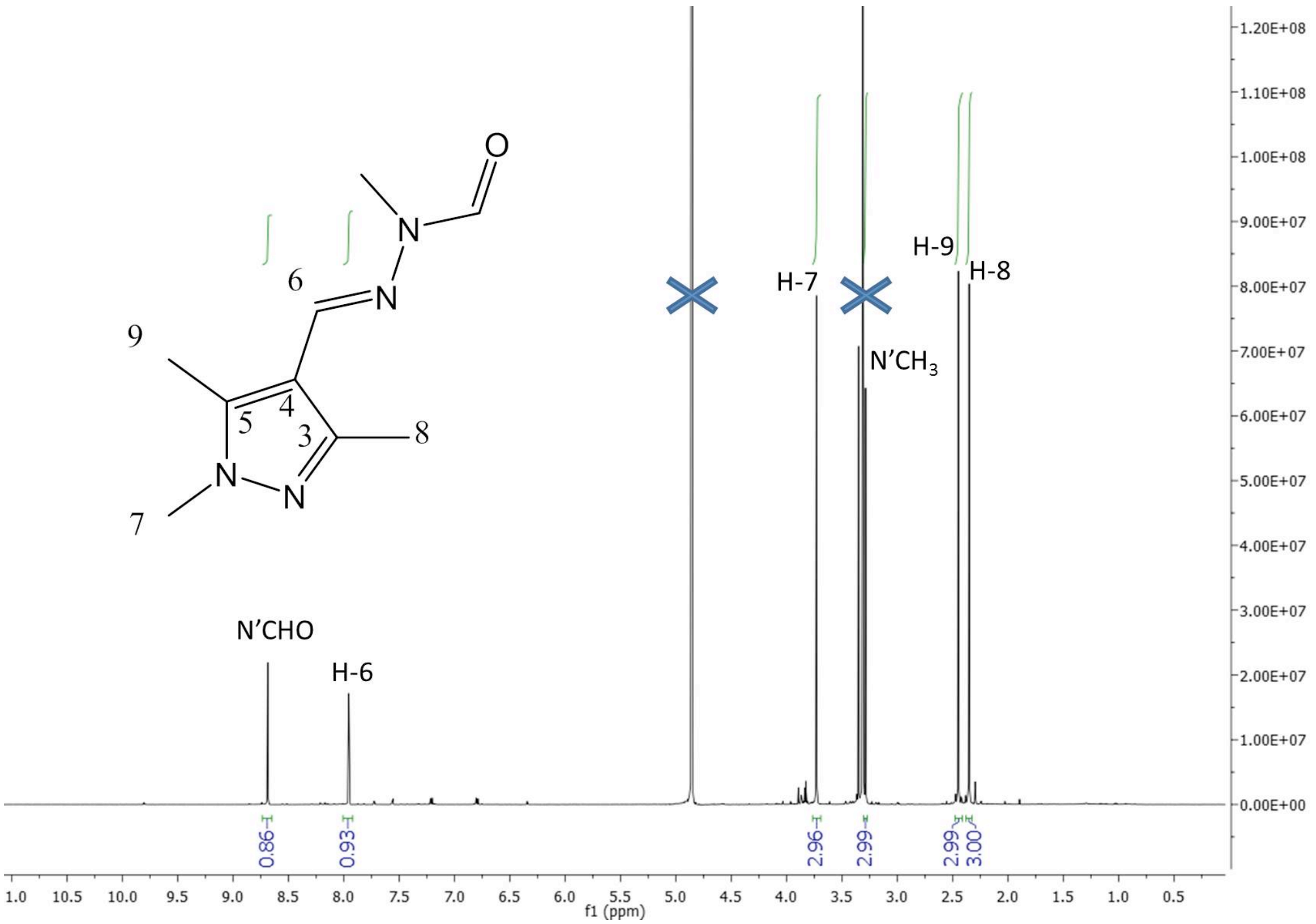

S7-1. ^{1}H NMR (MeOH-*d4*, 600 MHz) spectrum of **7**

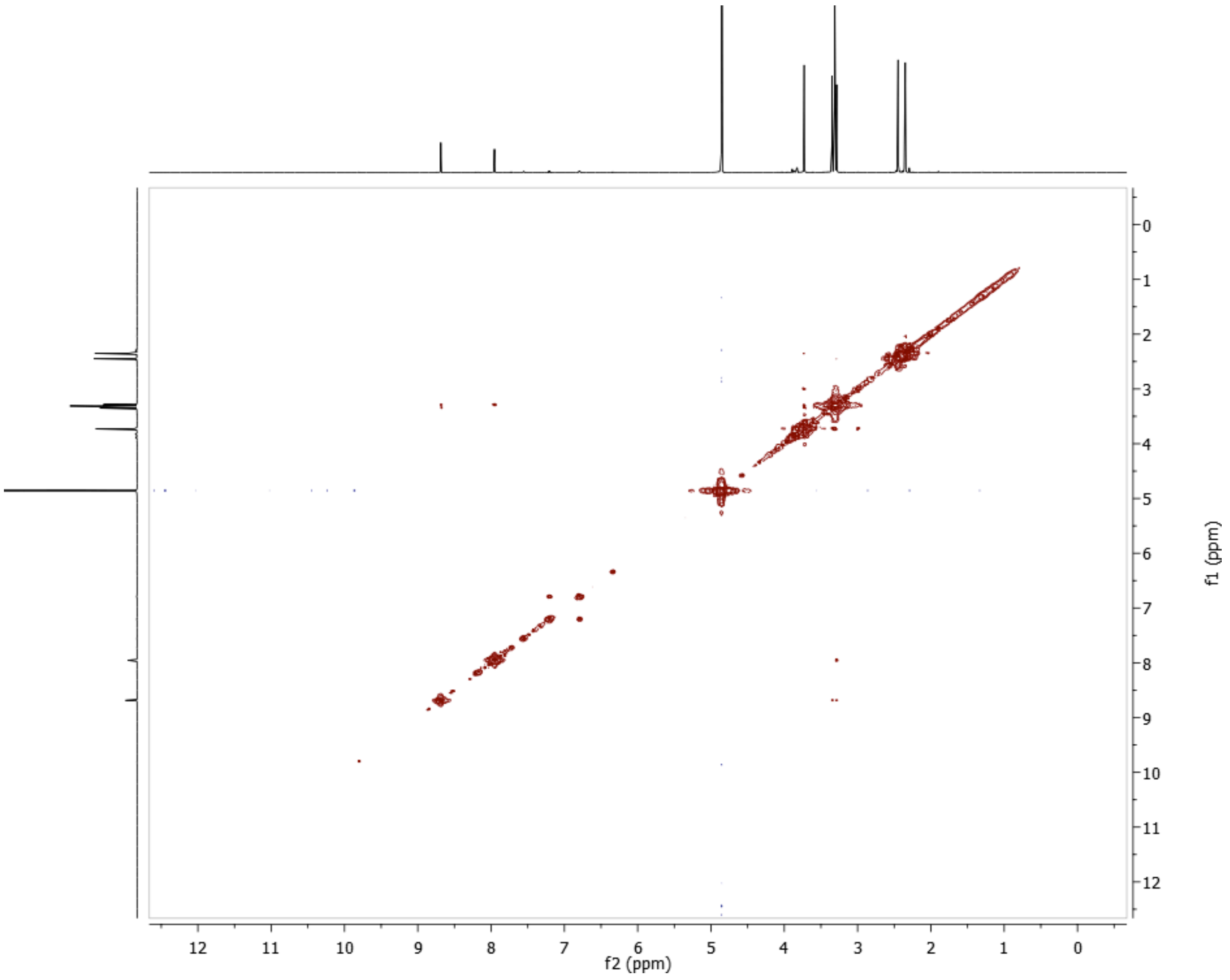

S7-2. COSY NMR (MeOH-d_4, 600 MHz) spectrum of **7**

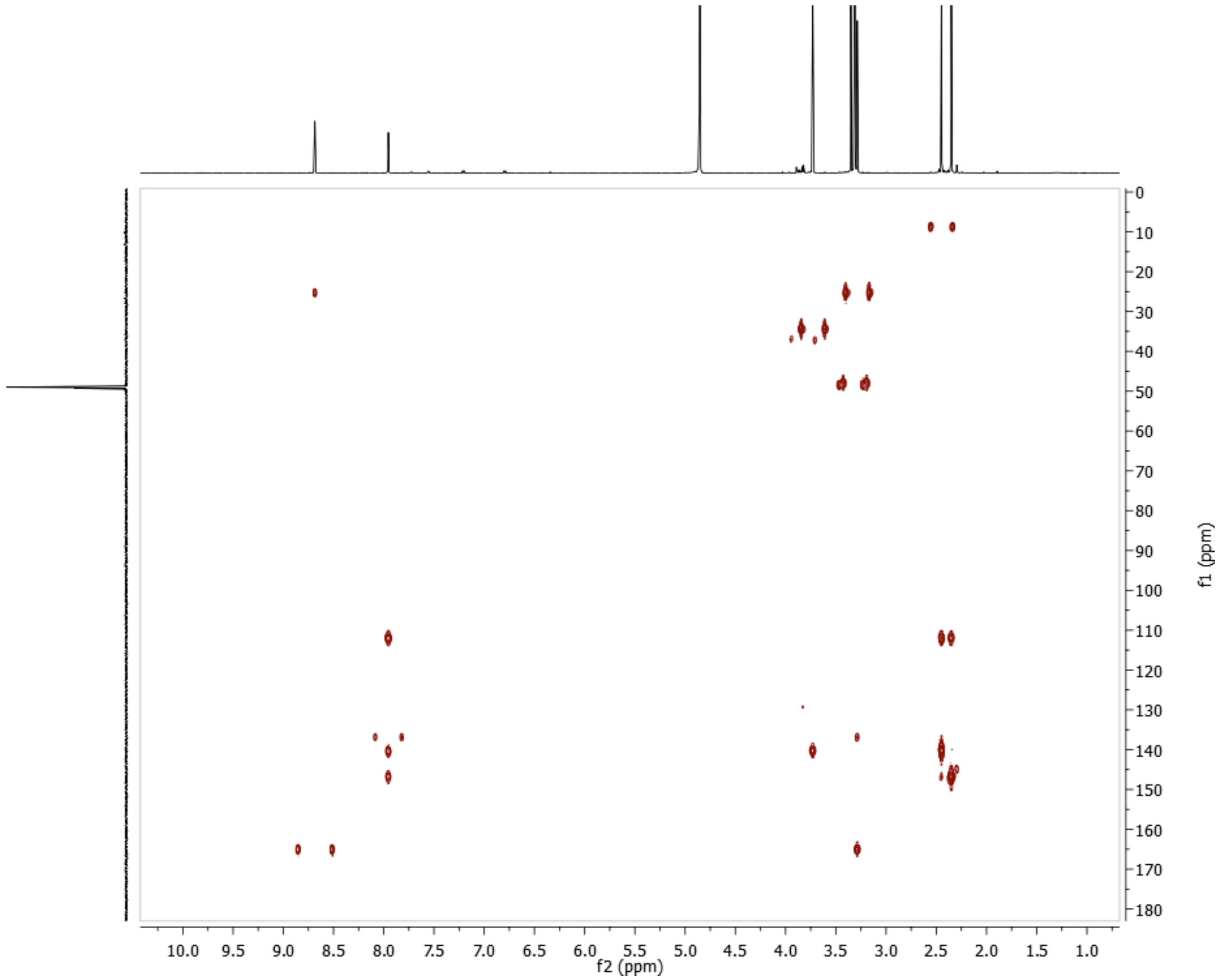

S7-3. HMBC NMR (MeOH-d_4, 600 MHz) spectrum of **7**

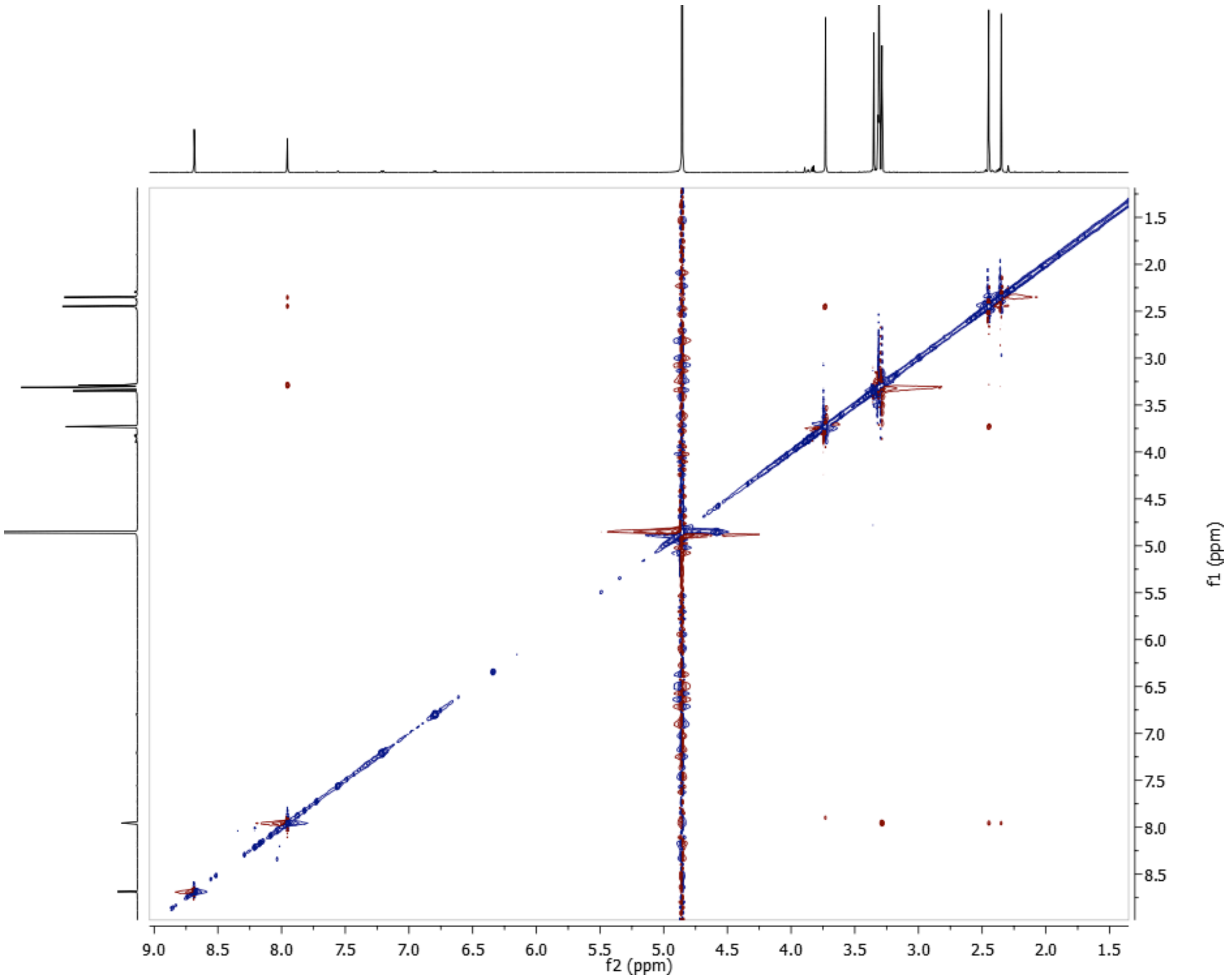

S7-4. ROESY NMR (MeOH-d_4, 600 MHz) spectrum of **7**

Mass Spectrum SmartFormula Report

Analysis Info

Analysis Name	D:\Data\spektren 2017\Proksch17HR000006.d	Acquisition Date	1/4/2017 3:09:42 PM
Method	tune_low_new.m	Operator	Peter Tommes
Sample Name	Amin Mokhlesi C2E5-3 (CH3OH)	Instrument	maXis 288882.20213
Comment			

Acquisition Parameter

Source Type	ESI	Ion Polarity	Positive	Set Nebulizer	0.3 Bar
Focus	Not active	Set Capillary	4000 V	Set Dry Heater	180 °C
Scan Begin	50 m/z	Set End Plate Offset	-500 V	Set Dry Gas	4.0 l/min
Scan End	1500 m/z	Set Collision Cell RF	600.0 Vpp	Set Divert Valve	Source

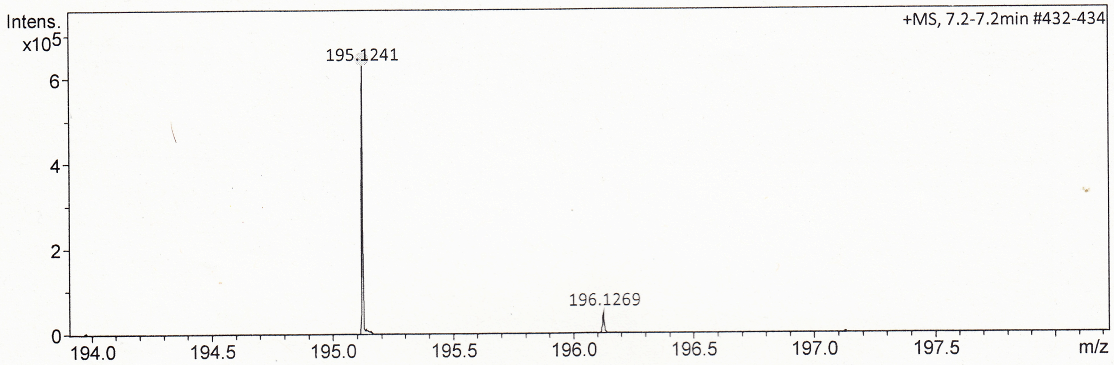

Meas. m/z	#	Ion Formula	m/z	err [ppm]	mSigma	# mSigma	Score	rdb	e⁻ Conf	N-Rule
195.1241	1	C8H19O5	195.1227	-7.3	7.8	1	62.61	-0.5	even	ok
	2	C9H15N4O	195.1240	-0.4	19.8	2	100.00	4.5	even	ok

S7-5. HRESIMS spectrum of **7**

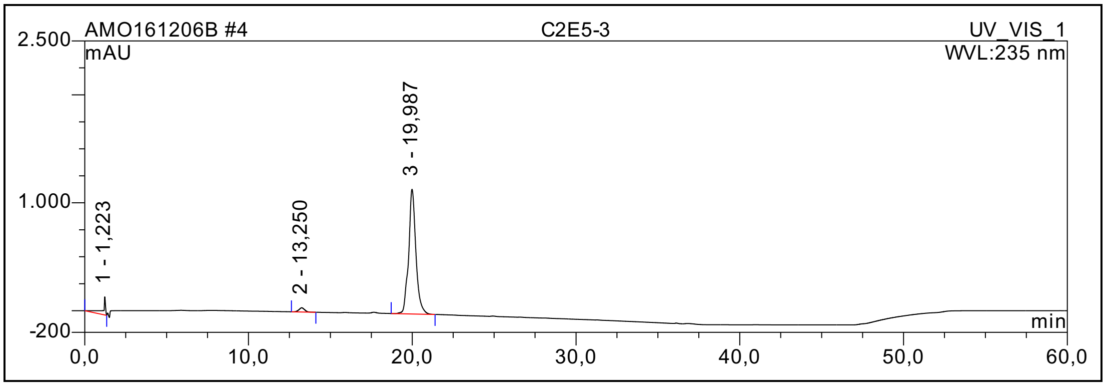

S7-6. HPLC chromatogram of **7**

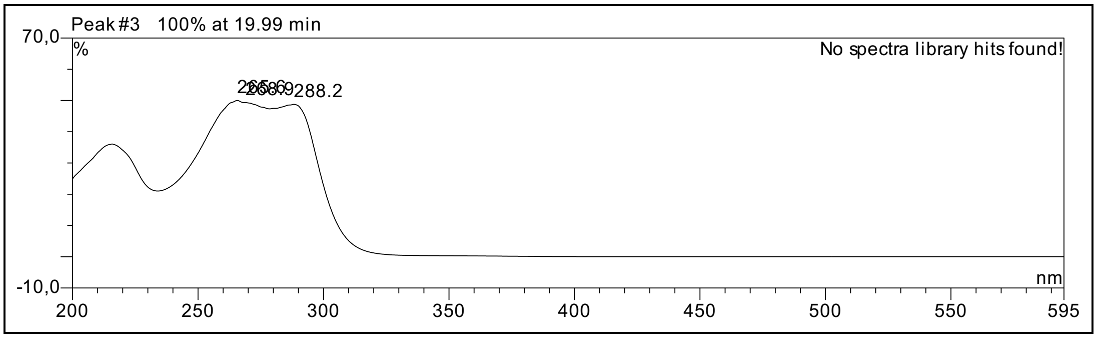

S7-7. UV spectrum of **7**
